# The Structures and Compositions Design of the Hollow Micro–Nano-Structured Metal Oxides for Environmental Catalysis

**DOI:** 10.3390/nano14141190

**Published:** 2024-07-12

**Authors:** Jingxin Xu, Yufang Bian, Wenxin Tian, Chao Pan, Cai-e Wu, Leilei Xu, Mei Wu, Mindong Chen

**Affiliations:** 1 State Key Laboratory of Low-Carbon Smart Coal-Fired Power Generation and Ultra-Clean Emission, China Energy Science and Technology Research Institute Co., Ltd., Nanjing 210023, China; xjx_0718@163.com (J.X.); twxtim@gmail.com (W.T.); 2 Collaborative Innovation Centre of the Atmospheric Environment and Equipment Technology, School of Environmental Science and Engineering, Nanjing University of Information Science & Technology, Jiangsu Key Laboratory of Atmospheric Environment Monitoring and Pollution Control, Nanjing 210044, China; bianyufang@emails.bjut.edu.cn; 3 College of Light Industry and Food Engineering, Nanjing Forestry University, Nanjing 210037, China; wucaie@njfu.edu.cn; 4 National & Local Joint Engineering Research Center for Mineral Salt Deep Utilization, Huaiyin Institute of Technology, Huaian 223003, China; 5 School of Environment and Energy Engineering, Anhui Jianzhu University, Hefei 230009, China

**Keywords:** hollow-structured metal oxides, environmental catalysis application, structures and compositions, structure–performance correlation

## Abstract

In recent decades, with the rapid development of the inorganic synthesis and the increasing discharge of pollutants in the process of industrialization, hollow-structured metal oxides (HSMOs) have taken on a striking role in the field of environmental catalysis. This is all due to their unique structural characteristics compared to solid nanoparticles, such as high loading capacity, superior pore permeability, high specific surface area, abundant inner void space, and low density. Although the HSMOs with different morphologies have been reviewed and prospected in the aspect of synthesis strategies and potential applications, there has been no systematic review focusing on the structures and compositions design of HSMOs in the field of environmental catalysis so far. Therefore, this review will mainly focus on the component dependence and controllable structure of HSMOs in the catalytic elimination of different environmental pollutants, including the automobile and stationary source emissions, volatile organic compounds, greenhouse gases, ozone-depleting substances, and other potential pollutants. Moreover, we comprehensively reviewed the applications of the catalysts with hollow structure that are mainly composed of metal oxides such as CeO_2_, MnO_x_, CuO_x_, Co_3_O_4_, ZrO_2_, ZnO, Al_3_O_4_, In_2_O_3_, NiO, and Fe_3_O_4_ in automobile and stationary source emission control, volatile organic compounds emission control, and the conversion of greenhouse gases and ozone-depleting substances. The structure–activity relationship is also briefly discussed. Finally, further challenges and development trends of HSMO catalysts in environmental catalysis are also prospected.

## 1. Introduction

With the occurrence of the global industrial revolutions and subsequent development of the economy, huge amounts of fossil fuels, such as naphtha and coal, have been consumed. As a result, a large number of atmospheric and aquatic environmental pollutants, such as carbon monoxide (CO), nitrogen oxides (NO_x_), etc., have been discharged into the environment and are harmful to the health of human beings [1,2]. The removal of these pollutants via environmental catalysis strategy is therefore of great importance from the viewpoint of environmental protection.

Broadly speaking, all the catalytic processes reducing and removing pollutant emissions and recycling resource utilization of the waste can be ascribed to the category of environmental catalysis, such as the below conditions: (a) eliminating the atmospheric, water, and indoor pollutants (e.g., CO [3], SO_x_ [4], NO_x_ [5], formaldehyde [2], toluene [6] and 4-Nitrophenol [7], etc.); (b) reducing the harmful substances generated in the energy conversion processes (e.g., supported metal catalysts are used to reduce polycyclic compounds and the biphenyls produced by the pyrolysis of industrial waste plastics) [8]; (c) converting the waste into useful resource [9]. The development of the efficient catalysts is considered the key factor of environment catalysis. In recent decades, structural engineering has attracted global widespread attention, and bridges the interplay between properties and performance.

At present, noble metals and transition metal oxide-supported catalysts are widely used in all kinds of environment catalytic processes [10]. Moreover, studies of catalysts have indicated that the catalytic activities of removing pollutants have been greatly related to various factors, such as the properties of the supports (e.g., specific surface area, porous structure, lattice oxygen mobility, etc.), the characteristics of the active centers (e.g., morphology, crystal face, dispersion, etc.), and the metal–support interaction (e.g., the adsorption of oxygen species, low-temperature reducibility, the ability to activate the reactants, the interaction between metals, etc.) [11].

Therefore, the catalytic ability could improve by adjusting the morphology, spanning from the atomic scale to the microarchitecture, and the spatial organization of components of the support [12,13]. As for the hollow micro–nano structured materials, they are defined as a type of functional nanomaterials with void spaces inside different shells [14]. In addition, the scarcity and cost of materials are also considered to be important concerns when developing new catalysts. The outstanding interfacial properties and higher atom utilization efficiency of HSMOs can be envisaged in potential catalysis application. Therefore, it is reasonable to design and construct novel catalysts with hollow structures in the viewpoint of atom economy.

With the in-depth study of hollow micro–nano structures, the HSMOs have received more and more attention in the field of environmental catalysis. HSMOs have advantageous physical properties such as high specific surface areas, tunable pore sizes, synergistic interaction, adjustable morphology, space utilization, abundant defects, adjustable surface chemistry, and low density, etc. Additionally, further advantages, such as high loading capacity, good surface penetration, water resistance, strong metal–support interaction (SMSIs), and magnetism, can be achieved by controlling their structure and composition. As a result, these have been widely used in many environmental catalysis reactions, including methane combustion [15], water–gas shift [16], CO oxidation [17], CO conversion [18,19], toluene oxidation [20,21], chlorinated aromatic compound oxidation [22], formaldehyde oxidation, etc. [23]. The summary of the application of HSMOs in the elimination of environmental pollutants is shown in Table 1.

Furthermore, HSMOs are also widely used in lithium-ion batteries [24,25], gas sensors [26], energy-related systems [27], and heterogeneous catalysts, etc. [28,29,30]. Recently, some excellent reviews have given comprehensive descriptions and discussions of HSMOs, showing their fascinating performance, fabrication, and properties [31,32,33,34]. However, these reviews did not comprehensively cover the composition and structure design of HSMOs or their applications in the field of environmental catalysis. Therefore, this review summarizes the different aspects of the application of HSMOs to environmental catalysis. In light of this, the synthetic strategies are only briefly introduced in this review. These strategies can be classified into four different types, including hard-templating, soft-templating, self-templated, and template-free methods. Therefore, this review will mainly focus on the recent progresses in the application of the HSMOs as the efficient catalysts and/or supports in the field of environmental catalysis, which performed lots of superiorities compared with the traditional counterparts. To highlight the catalytic performance of HSMOs, we focus on the catalytic activity as the main performance descriptors rather than the stability and selectivity.

## 2. The Application of HSMO Catalysts in Environmental Catalysis

### 2.1. Automobile and Stationary Sources Emission Control

The untreated exhaust gases of automobiles, chemical plants, and coal-fired power plants such as CO, NO_x_, SO_x_, and other harmful gases, have caused serious environmental problems and human health issues [35,36]. As it is well known, CO is a toxic atmospheric pollutant that is both flammable and explosive, whilst NO_x_ and SO_x_ can the cause acid rain and photochemical smog, which negatively affect human respiratory system. In order to alleviate the related pollutants’ emissions, various strategies, such as adsorption, absorption, catalytic oxidation, incineration, plasma destruction, and photocatalysis, etc., have been widely investigated. It is clear that catalytic oxidation has been considered to be the most effective method because of its unique advantages, such as its high efficiency and cleanliness [36,37]. Therefore, lots of efforts have been devoted to the development of efficient catalysts to control the emissions of automobile and stationary sources. Meanwhile, HSMO-based catalysts provide promising and valuable chances to develop advanced catalysts due to the advantages of HSMOs in terms of their low density, large surface-to-volume ratios, reduced mass transport length, and high loading capacity.

#### 2.1.1. Catalytic Oxidation of CO

Industrial and automobile CO emissions have been increasing year upon year. The removal of CO emissions has become an important concern because of their high toxicity to human health and the living environment. The catalytic oxidation of CO has been considered the most effective treatment method [10]. The HSMOs of various structures have been used to catalyze CO oxidation in the past two decades. The hollow interior space of HSMOs is expected to effectively reduce the density of the material and enhance the permeability of the material. As a result, they can provide gaseous reactants with large specific surface areas for the absorbance and mass transference of the CO molecules to the active center. In addition, the extended contact time between CO molecules and the active center have potentially positive impacts on the whole catalytic process.

The ‘lattice oxygen’ mechanism believes that the oxygen supply ability of metal oxides is a key factor influencing catalytic reactions [38]. To date, numerous catalysts have been investigated in the preliminary study of CO oxidation. Among these catalysts, Ceria (CeO_2_), with its cubic fluorite structure, has been considered a key promoter of catalytic CO oxidation. Therefore, the catalysts for CO oxidation are divided into the hollow micro/nano-structured CeO_2_-based materials (HMNCMs) and other HSMOs and highlighted in this subsection. For HMNCMs, CeO_2_ performs variable oxidation states, and has good redox properties and a high storage/release oxygen capacity thanks to its abundant oxygen vacancy, the redox property of Ce^3+^/Ce^4+^, and its structural integrity [39]. Studies of CeO_2_ morphology control for the catalytic oxidation of CO can be traced back to 2006 [40]. Lots of studies have focused on the shape-controlled synthesis of Ce-based nanomaterials and their corresponding catalytic applications [41,42]. Ce-based nanomaterials can obtain a controllable morphology through the reasonable regulation of reaction conditions [43]. To date, many Ce-based catalysts with hollow structures have been successfully designed and fabricated [44,45,46,47,48,49,50,51,52], which accelerate the process of their practical application [53]. However, there is no complete report on the application of HMNCMs toward the catalytic CO oxidation.

It is worth noting that lots of HMNCMs with different compositions and morphologies have been developed since 2012 [54,55,56,57,58]. The HMNCMs could be divided into the pure CeO_2_ hollow structure, the composite binary or multiple CeO_2_ hollow structures, the multi-element Ce-based hollow structure, and the Ce-based hollow structure-doped with noble metals according to the composition. The critical factors of reaction conditions, the possible formation mechanism on the morphology and assembly of the HMNCMs, and the CO oxidation catalytic activities have been investigated in previous studies. The amazing progress of hollow micro/nano-structures have been largely driven by the development of analytical technologies and the simultaneous development of template materials [32]. Therefore, in order to clearly prospect and outlook the development and changes, the related information and details about HMNCMs and other HSMOs were summarized.

- Pure CeO_2_ hollow structure

The previous investigation into CeO_2_ with a hollow structure morphology revealed that the exposed special crystal facets, small CeO_2_ crystal sizes, and a significantly deformed structure in the boundary area were key factors for the improved CO oxidation activity [50]. Additionally, the oxygen storage capacity of CeO_2_ is greatly related to the morphology or surface structure [45,59]. Therefore, it is of great necessity to explore and develop CeO_2_ with hollow structure. Studies have shown that {100} surface ceria nanocrystals performed a higher CO oxidation activity than those with {111} surface-dominant [60,61], which was related to the lattice oxygen migration of {100}/{110}-dominated surface structures [59,62]. Han et al. [63] fabricated the CeO_2_ hollow structure catalyst with a significant improvement in the catalytic CO oxidation activity. The reason for this was that the CeO_2_ hollow structure exposed more {001} faces, which have more dangling bonds on the surface and internal Ce atoms. Therefore, the CeO_2_ with abundant dangling bonds, mesoporosity, oxygen vacancies, and high surface areas were beneficial to their catalytic performance in CO oxidation.

There are also other pure CeO_2_ hollow structures, including CeO_2_ hollow nanocones [64], CeO_2_ hollow microspheres [65,66,67], and CeO_2_ hollow dodecahedrons [68]. A summary of pure CeO_2_ with various hollow structures is presented in Table 2. Among them, Li et al. [68] found that the CO catalytic activity of the cracked hollow CeO_2_ dodecahedrons was significantly lower than that of the hollow CeO_2_ dodecahedrons due to the presence of oxygen vacancy defects and the permeability of the shell. The hollow CeO_2_ dodecahedrons and the CeO_2_ hollow nanocones exhibited excellent CO catalytic activity, which was ascribed to the homogeneously dispersed particles with mesoporous structures comprising the highly specific BET surface area morphological features. However, the catalytic activity of these CeO_2_ hollow structures still does not meet the requirements of industrial applications because the hollow catalyst possesses the disadvantage of a structural collapse as well as low activity. Therefore, CO oxidation catalysts require a stable structure to achieve excellent catalytic stability.

Furthermore, compared with other hollow-structured CeO_2_, hollow CeO_2_ dodecahedrons performed the best CO oxidation catalytic activity because of the incorporation of Co species [68]. Although the CeO_2_ itself cannot achieve a high catalytic CO oxidation activity, it can incorporate other metals as structural and/or electronic promoters to improve its catalytic activity and stability with the priority of not destroying the hollow structure. Based on these basic studies, HMNCMs doped with transition metals have been further developed.

- The composite binary or multiple CeO_2_ hollow structure

It was reported that the synergistic effect created by incorporating the transition metal (Co, Cu, Mn, Ni, and Fe) into CeO_2_ could greatly improve the catalytic activity [70,71,72,73]. In Table 3, we summarize the composite binary or multiple CeO_2_ materials with various compositional, hollow structures, and the catalytic parameters for CO catalytic oxidation. The changes in the physical properties of hollow catalysts with different structures will increase the catalytic CO oxidation activity. For example, the Co_3_O_4_-CeO_2−x_ hollow multi-shell structure (HOMS) could achieve the complete conversion at 166.9 °C, while the complete conversion temperature of Co_3_O_4_-CeO_2−x_(Co/Ce = 4/1) nanoparticles (NPs) was 206 °C [70]. For hollow mesoporous Co_3_O_4_-CeO_2_ composite nanotubes with open-ends, the non-closed structure can accelerate the reactants to enter the hollow structure of the catalyst to contact more active centers, accounting for the 100% CO conversion at 145 °C [71].

**Figure 1:**
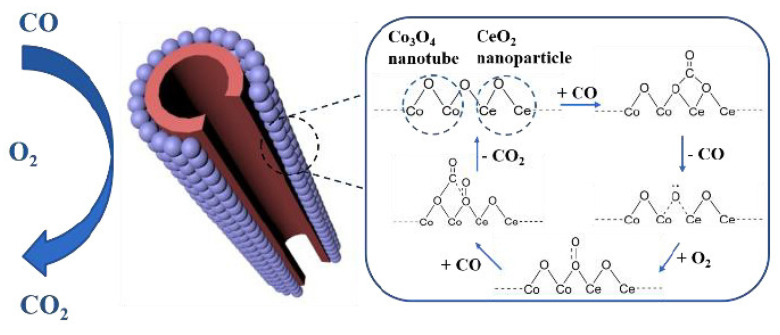
Schematic of the reaction mechanism for CO oxidation. Figure reproduced form ref. [71].

As shown in Figure 1, the Co catalytic CO oxidation mechanism was addressed by the Langmuir–Hinshelwood (L-H) model, which contained the four following steps [71]: (1) The CO was adsorbed into the interface of Co_3_O_4_ and CeO_2_. (2) The CO_2_ molecule was formed with the oxygen vacancy left due to the extraction of surface oxygen by CO. (3) The O_2_ reacted with the oxygen vacancy and the amount of adsorbed O_2_ increased, with the dissociation of O_2_ into the O_2_^−^ ion radical, which promoted the enhancement of the CO oxidation. (4) The O_2_^−^ ion radical reacted with the CO molecule and the CO_2_ molecule was formed. The oxygen vacancy was a vital part for the dissociation of O_2_ in the CO oxidation reaction, which linked with the metal nanoparticles size, the oxidation states, and the oxidation states of the catalyst.

As a new type of composite materials, the hollow composite materials have attracted increasing attention. Liu et al. fabricated the CeO_2_-CuO_x_ hollow nanospheres with loos and a rough surface, which demonstrated that they had a more unique structure because Cu species were more inclined to concentrate on the surface of CeO_2_ hollow spheres compared with the pristine CeO_2_ [74]. In addition, the Cu species was the active site of CeO_2_-CuO_x_ composite hollow spheres in terms of catalytic CO oxidation [75]. The structural stability of the CuO_x_/CeO_2_ interface is also a very important concern for Cu-doped CeO_2_ catalysts. The sintering of the surface CuO_x_ during the reaction often causes changes in the surface copper species and crystal structure of cerium oxide, which in turn affect the catalytic activity.

The active center theory believes that the corners, steps, edges, dislocations, defects, and other discontinuities on the catalyst surface would modify the nature of the adsorbed species and the dynamics of the surface reactions [88]. Their catalytic activities are usually higher than those on a flat surface. As a result, these sites are considered active centers. As shown in Table 3, abundant steps were exhibited on the surface of the novel litchi-peel-like hierarchical hollow copper–ceria microspheres, which was crucial for the improvement in catalyst activity [76]. The excellent catalytic activity could be attributed to the step-stabilized strong interaction between CuO_x_ species and CeO_2_ and the abundant surface steps in litchi-peel-like samples that act as adsorption sites for oxygen.

Many researchers believed that the copper–ceria catalyst was a sort of promising alternative that could substitute for noble metal catalysts due to its low cost and decent catalytic activity [78]. Therefore, the spiny yolk@shell CuO@CeO_2_ cubes with a hedgehog-like surface composed of large spiny CuO crystal whiskers [77], triple-shelled CuO/CeO_2_ hollow nanospheres [79], and hollow-multiporous wall CeO_2_-supported CuO catalysts [78] were prepared. The hollow-multiporous wall CeO_2_ supported CuO catalysts for CO oxidation with a T_100_ of around 60 °C. However, its stability test was not so excellent compared to other Cu-doped CeO_2_ catalysts.

It is common practice to improve the catalytic activity of HMNCMs by incorporating Mn. Zhang et al. [82] prepared MnO_2_@CeO_2_–MnO_2_ composite hollow spheres exhibiting a superior catalytic performance by the facile three-step method, which employed carbon spheres (CSs) as sacrificial templates. Chen et al. prepared CeO_2_–CuO with a core–shell structure [80] and porous/hollow-structured CeO_2_-MnO_x_ [84] to promote the performance of the catalytic CO oxidation. Liu et al. [81] fabricated CeO_2_–MO_x_ (M = Cu, Co, Ni) composite yolk–shell nanospheres by the general wet-chemical approach. After this, they fabricated a series of MCo_2_O_4_@CeO_2_ (M = Ni, Cu, Zn, Mn) core@shell nanospheres [80], double-shelled Fe_2_O_3_/CeO_2_ boxes [87], CeO_2_@MnO_2_ core@shell nanospheres [83], Mn_2_O_3_@CeO_2_ core@shell cubes [85], and CeO_2_-MnO_x_ hollow 3D porous architecture [86].

Obviously, the doped metal Cu of Ce-based martials played a pivotal roles in catalytic reaction. However, the comparison of catalytic activities toward the CO oxidation of the and hollow-multiporous wall CeO_2_ supported CuO catalysts and the CeO_2_–CuO_x_ hollow nanospheres indicated that the adjustable morphology of catalysts endows the catalyst to rationalize a multitude of factors, such as the synergistic interaction, high surface area, space utilization, high loading capacity, superior pore permeability, and abundant inner void space.

- The multi-element Ce-based hollow structure

**Figure 2:**
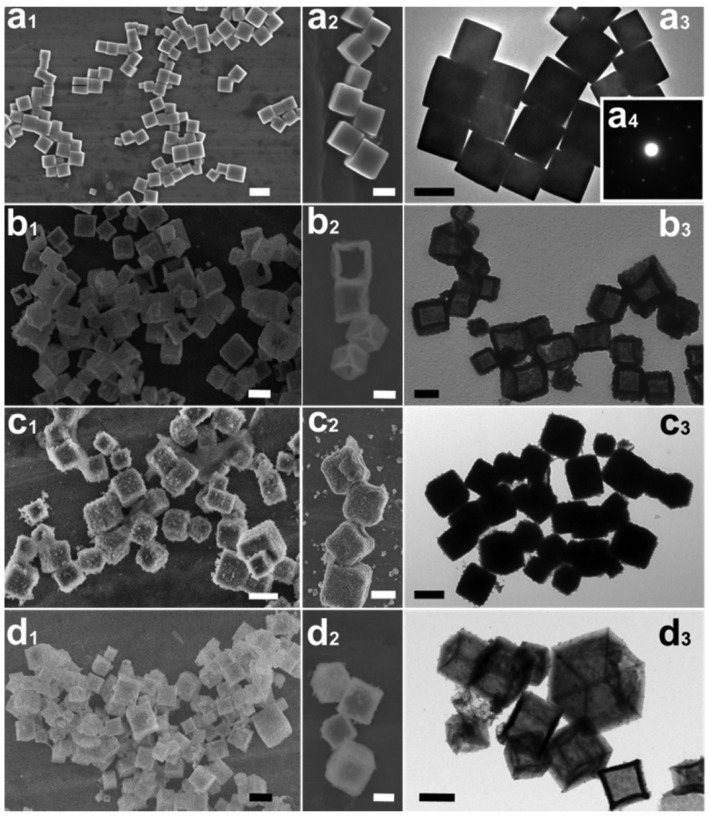
The SEM (x1 and x2) and TEM (x3) images of the Cu_2_O cubes, composite CeO_2_-Cu_2_O (1), NiO@Cu_2_O (2), and CeO_2_–NiO–Cu_2_O (3). (x = a, b, c and d for Cu_2_O and composites 1−3, respectively) The scale bar is 800 nm in parts x1 and x3, and it is 500 nm in part x2. Figure reproduced from ref. [89]. (**a_1_**,**a_2_**) The SEM images of Cu_2_O cubes; (**a_3_**) The TEM images of Cu_2_O cubes; (**a_4_**) The SAED pattern of Cu_2_O cubes; (**b_1_**,**b_2_**) The SEM images of composite CeO_2_-Cu_2_O; (**b_3_**) The TEM images of composite CeO_2_-Cu_2_O; (**c_1_**,**c_2_**) The SEM images of composite NiO@Cu_2_O; (**c_3_**) The TEM images of composite NiO@Cu_2_O; (**d_1_**,**d_2_**) The SEM images of composite CeO_2_–NiO–Cu_2_O; (**d_3_**) The TEM images of composite CeO_2_–NiO–Cu_2_O.

Additionally, multi-metal doped Ce-based CO oxidation catalysts have also been extensively studied. Some parameters of the hollow structure, such as the composition and wall thickness, are related to the synergy between metals. Liu et al. [89] reported the hollow CeO_2_-=–Cu_2_O, core–shell NiO@Cu_2_O, and hollow CeO_2_-NiO-Cu_2_O cages (Figure 2). The multicomponent metal oxides with a hollow structure exhibited a lower CO oxidation temperature than Cu_2_O cubes.

Cheng et al. [90] synthesized NiCo_2_O_4_@CeO_2_ core@shell nanotubes with a tunable shell thickness through the layer-by-layer coating method and employed these materials as the high-performance catalyst of the CO oxidation reaction. The NiCo_2_O_4_@CeO_2_-2 (2 represented the molar ratio of Ce/(Ni + Co)), which showed the highest catalytic activity: over 50% of CO can be oxidized at a low temperature of 100 °C, and the final T_100_ (100% conversion temperature) was about 150 °C. The effect of different shell structures on catalytic performance was studied. The results showed that the two-phase interface area of NiCo_2_O_4_@CeO_2_-1 with the thinnest CeO_2_ shell will decrease, which weakens the synergistic effect between CeO_2_ and NiCo_2_O_4_.

- Ce-based hollow structure doped with noble metals

Huge challenges such as the low thermal stability and loss of catalytic activity due to sintering still need to be solved in catalytic applications. This was attributed to the noble metal nanoparticles which tend to aggregate during the process of catalytic reaction, causing the rapid decay of catalytic activity and stability [12]. The decentralized function of HMNCMs is a critical advantage for noble metal materials. Thus, designing and fabricating HMNCMs to suppress the aggregation and sintering of noble metal nanoparticles is an effective and promising solution [63,91]. The summary of the Ce-based hollow structure doped with noble metals is presented in Table 4.

The noble metal NPs act as the active catalytic sites for CO oxidation. The role of hollow ceria as support is primarily to stabilize noble metal NPs to prevent their sintering during catalytic reactions. In addition, ceria also works as an electronic modulator for the loaded noble metal NPs [92,95,97]. The interior void space of hollow CeO_2_–ZnO microspheres fabricated by Xie et al. [94] can be clearly observed in Table 4. The Ce–OZn linkages formed at the interface between CeO_2_ and ZnO nanoparticles, which was conducive to strengthening interfacial interactions and CO adsorption. Consequently, the catalytic activity of CO oxidation over the CeO_2_–ZnO composite hollow microspheres was greatly improved due to the synergistic effect between CeO_2_ and ZnO. They loaded the Au nanoparticles on the surfaces of the CeO_2_–ZnO composite hollow microspheres by the deposition−precipitation method to further improve the CO oxidation catalytic activity.

Many HMNCMs have been used as the support for Au nanoparticles, such as Au@CeO_2_-ZrO_2_ with a hollow core–shell structure [98], Au/CeO_2_ hollow nanospheres [3], Au/CeO_2_ nanotubes [99], and sandwich hollow-structured CeO_2_@Au@CeO_2_-MnO_2_ [103]. Compared to non-hollow-structured core–shell Au@CeO_2_ nanocomposites [104], these hollow-structured catalysts exhibited a higher catalytic activity for CO oxidation.

**Figure 3:**
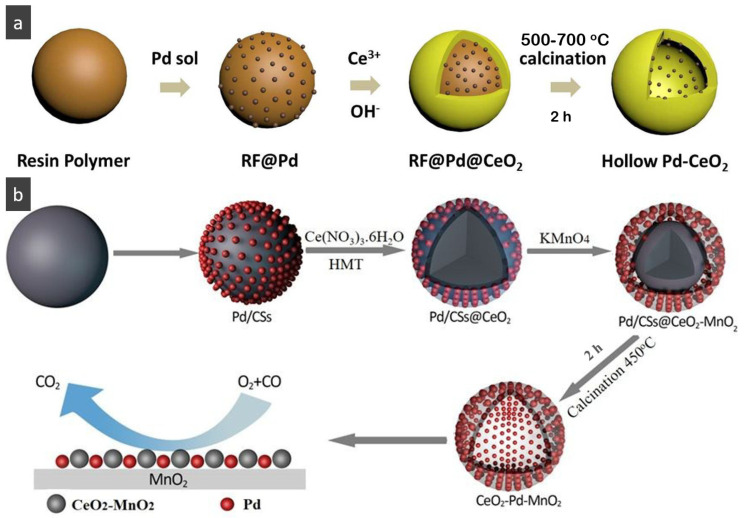
(**a**) The formation process of the hollow Pd–CeO_2_ nano-composite sphere reproduced from ref. [91]; (**b**) Schematic illustration of the synthesis process for sandwich-like MnO_2_–Pd–CeO_2_ hollow spheres. Figure reproduced from ref. [93].

HMNCMs loaded with Pd can effectively prevent particle migration and deactivation by separating precious metal NPs in the small cavities [91]. As shown in Figure 3a, (1) Pd NPs were fully deposited on the surface of RF polymer spheres to form RF@Pd structure; (2) RF@Pd particles were exposed to the solution containing Ce^3+^ and hexamethylenetetramine (HTMA) to form RF@Pd@CeO_2_; (3) Hollow Pd–CeO_2_ nano-composite spheres (NCSs) were fabricated by calcination to eliminate the polymer templates.

For other HMNCMs doped with metallic Pd NPs, Zhang et al. [93] prepared sandwich-like MnO_2_-Pd-CeO_2_ hollow spheres by depositing Pd nanoparticles on the outer surface of the MnO_2_ shell, before coating it with CeO_2_, which had an anchoring effect on the outermost layer (Figure 3b). The hollow spheres exhibited excellent stability and CO oxidation activity due to the sandwich structure and the strong synergy between Pd and the layered porous MnO_2_–CeO_2_ shell.

There are also many works which have incorporated metal Pt NP into HMNCMs to improve their catalytic activity. Wang et al. [100] found that Pt cations on the CeO_2_–Pt interface could make the Pt–CO bond weak, which made the reduction in oxygen easy during the CO oxidation reaction. Additionally, the CeO_2_–Pt nanotube composites also had excellent thermal stability, even when calcined at a high temperature up to 700 °C. This demonstrated that the hollow structure could prevent the migration and sintering of Pt NPs. Similarly, the CeO_2_ hollow sphere embedded with Pt (Pt/CeO_2_ HS) [101] and Pt/CeO_2_@SiO_2_ catalysts with the porous/hollow structure [102] exhibited high activity and excellent durability in terms of CO oxidation. The significant increase in the CO conversion rate was attributed to the presence of internal voids in the material, which enhanced the chemisorption of CO on the Pt sites.

- Other HSMOs

The rational morphological design and component optimization of other metal oxides doped in HMNCMs such as Fe_2_O_3_, Co_3_O_4_, CuO, etc., have also been studied. Based on this, other HSMOs applied to CO oxidation are summarized in Table 5.

As displayed in Table 5, Li et al. [66] prepared the hollow transition metal oxide microspheres (CeO_2_, α-Fe_2_O_3_, and Co_3_O_4_) via a general ultrasonic-spray-assisted synthesis method for catalytic CO oxidation. The catalytic activity expressed by the CO conversion of these hollow/mesoporous transition metal oxide microspheres followed the following sequence: Co_3_O_4_ hollow microspheres > CeO_2_ hollow microspheres > α-Fe_2_O_3_ hollow microspheres. Therefore, Co_3_O_4_ was demonstrated to be an efficient catalyst for the oxidation of CO.

The other hollow-structured Co_3_O_4_ catalysts include the H–Co_3_O_4_@H–C (hollow Co_3_O_4_ NPs embedded in hollow carbon shell) [105], hollow and core–shell nanostructure Co_3_O_4_ [106]. The activities of these hollow-structured Co_3_O_4_ catalysts were improved by introducing hollow structures into the Co_3_O_4_ NPs. The hollow nanostructure Co_3_O_4_ provided more abundant active sites which are beneficial to the catalytic activity compared to the core–shell nanostructure Co_3_O_4_. However, the core–shell Co_3_O_4_ exhibited greater long-term stability than the hollow nanostructure Co_3_O_4_. This may be ascribed to the shell structure being prone to collapse without Co_3_O_4_ cores providing support points. Therefore, HSMOs could be designed with hollow structures to increase the oxygen vacancies and provide abundant active sites to improve the catalyst activity. The stability of the hollow structure could be increased by structural adjustment.

Moreover, second active metal oxides can be addicted to catalysts using metal–support interactions to improve the general catalytic performance. Zeng et al. [107] synthesized the Au/α-Fe_2_O_3_ catalysts with a varied hollow structure, which displayed the high catalytic performance of CO oxidation. Comparing α-Fe_2_O_3_ supports with the spindle, rod, and hollow rod structures, the hollow α-Fe_2_O_3_ nanoparticles exhibited the best activity due to the strong Au–support interaction with the Au nanoparticles.

**Figure 4:**
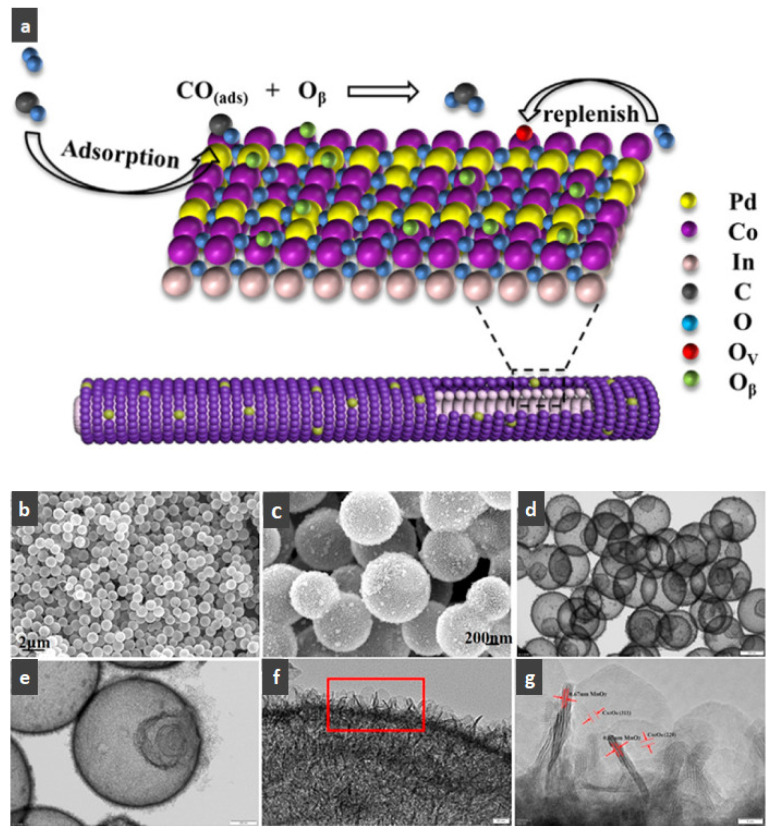
(**a**) The reaction mechanism of CO oxidation over the In_2_O_3_@Pd–Co_3_O_4_ catalyst reproduced from ref. [108]; (**b**,**c**) SEM images, (**d**–**f**) TEM images, and (**g**) HRTEM images of the MnO_2_–Co_3_O_4_ hollow spheres reproduced from ref. [109].

For hollow micro/nanostructured materials doped with metallic Pd NPs, Du et al. [108] fabricated the hollow In_2_O_3_@Pd–Co_3_O_4_ core/shell nanofiber catalyst with a higher CO oxidation activity (T_90_ = 56 °C) and lower activation energy. Additionally, they also confirmed that the ultra-thin shell structure and structural defects of In_2_O_3_@Pd-Co_3_O_4_ increased the redox capability. The high content of Pd^2+^, the small proportion of Co^3+^, and the increase in chemisorbed oxygen species were also possible reasons for the improvement in its catalytic performance. Moreover, the L-H mechanism could effectively explain the catalytic CO oxidation over the In_2_O_3_@Pd–Co_3_O_4_ catalyst. The possible CO oxidation reaction mechanism over the In_2_O_3_@Pd–Co_3_O_4_ catalyst is described in Figure 4a. Firstly, CO and O_2_ molecules were adsorbed onto the surface of the catalyst; secondly, Pd^2+^ and Co^3+^ activated the adsorbed CO molecules, and formed CO _(ads)_; then, the oxygen adsorbed on the surface was captured to convert CO _(ads)_ into CO_2 (ads)_; then, the CO_2 (ads)_ was converted into the CO_2_ and the oxygen vacancies of the sample were regenerated; finally, the O_2_ in the reactant supplemented the oxygen vacancies, and the absorbed oxygen was also regenerated. The Pd^2+^ is the main active sites for CO oxidation. According to the XPS measurement, the oxidation states of Pd were Pd^0+^ and Pd^2+^. The surface atomic ratio of Pd^2+^/(Pd^0+^ + Pd^2+^) was 51.6% and the In^3+^ oxidation state of In was presented in the catalysts.

As shown in Figure 4b–f, the Co–Mn composite hollow spheres [109] were successfully prepared through a ‘Kirkendall effect’ method. The unique surface structure of the MnO_2_–Co_3_O_4_ composite oxides could expose the abundant catalytic active sites of the interface between Co_3_O_4_ and MnO_2_ (Figure 4f). The Co_3_O_4_ nanoparticles with the ultra-thin nanosheet structure can be observed in Figure 4g, which could provide plentiful surface oxygen species and the strong adsorption ability of CO to improve the catalytic performance. The high content of Co^3+^ and Mn^4+^ facilitated the formation of oxygen vacancies in the catalysts. Therefore, the multi-shelled MnO_2_–Co_3_O_4_ hollow spheres exhibited a reliably high activity for CO oxidation due to its strong synergistic effect and the abundant oxygen vacancies between Co_3_O_4_ and MnO_2_.

Generally, traditional metal oxide-based catalysts can achieve acceptable CO catalytic activity at high temperatures, while noble metal-based catalysts can achieve them at relatively low reaction temperatures. However, the high cost of precious metal catalysts limits their availabilities and wide applications [10]. Therefore, it is both greatly necessary and urgent to develop the ‘noble-metal-free’ catalyst with the low-temperature catalytic performance of CO oxidation.

In summary, HSMOs were widely used to catalyze the CO oxidation reactions, while more attention was paid to HMNCMs due to the advantageous physical properties such as the high specific surface areas, big pore volumes, synergistic interaction, abundant defects, adjustable morphology, space utilization, and low density, etc. In addition, ceria is a very important oxygen storage material because of the reversible valence couple in Ce^4+^/Ce^3+^ and the high mobility of oxygen vacancies. There are generally two sources of oxygen vacancies in CeO_2_, specifically, the intrinsic oxygen vacancies and the foreign oxygen vacancies generated by doping the heteroatoms.

Firstly, the pure CeO_2_ hollow structure was considered a promising catalyst for CO oxidation. The improved CO oxidation activity of the pure CeO_2_ hollow structure depended on a critical factor, such as the exposed special crystal faces, small CeO_2_ crystal sizes, and significantly deformed structure in the boundary area. Afterward, with the growing tendency towards the fabrication of nanomaterials, more and more studies have focused on HMNCMs, including the composite binary, multiple CeO_2_ hollow structure, and Ce-based hollow structure doped with noble metals. Hereafter, the multi-element Ce-based hollow structure emerged only recently. CeO_2_ can generate a strong synergistic effect with other components in the catalytic process. Other HSMOs have similarly received a research process as HMNCMs. Therefore, the strong synergistic effect of transition metal oxides has been widely reported in the field of CO catalytic oxidation using the hollow structure of HSMOs to increase the exposed surface area of the active center to the reactants.

It is generally believed that catalysts with multi-shell hollow structures can obtain a better structure and catalytic stability than the single-shell counterpart because the multi-shell layers possess different supporting functions and the outer shell has a protective effect on the inside shell. Compared with single-shell hollow metal oxides, the multi-shell structure has a larger specific surface area, easier diffusion kinetics, higher bearing capacity, and lower density. Modifying the surface of the hollow structure by manufacturing defects, adding active metal components, and reducing the thickness of the hollow structure wall can greatly improve the catalytic activity. The hollow structure can greatly facilitate the internal diffusion of reactants and the external diffusion of products during the reaction [86]. In addition, increasing the number of shells of the hollow structure can improve the thermal stability of the hollow structure [110].

#### 2.1.2. NH_3_-SCR Removal of NO_x_

The emission of nitrogen oxides (NO_x_) is mainly derived from the high temperature combustion of fuel such as the coal combustion process in power plants and mobile transportation, or in stationary sources such as glass furnace and ceramics factory. [111] They constitute one of the main factors responsible for causing environmental problems, such as acid rain, ozone layer holes, and photochemical smog [112]. The elimination of NO_x_ air pollutants has become one of the public’s main concerns. To date, two methods have been applied to eliminate NO_x_ by HSMOs catalysts: the oxidation method and the low-temperature selective catalytic reduction of NO_x_ with NH_3_ (NH_3_-SCR) (Equations (1) and (2)).

4NO + 4NH_3_ + O_2_ → 4N_2_ + 6H_2_O, (1)

2NO_2_ + 4NH_3_ + O_2_ → 3N_2_ + 6H_2_O, (2)

In fact, the NO catalytic oxidation will enhance the SCR process via ‘fast’ SCR reaction: 4NH_3_ + 2NO + 2NO_2_ → 4N_2_ + 6H_2_O [113,114]. To be specific, the fast SCR reaction can be inspired by the partial oxidation of NO into NO_2_ [115,116]. In addition, a suitable NO/NO_2_ ratio is demanded for the oxidation method. However, the ratio of NO in the flue gas accounts for 95% of the NO_x_. Therefore, the catalytic oxidation of NO to NO_2_ plays an important role in the technique to eliminate NO_x_.

The hollow structure of the functional material has various advantages, such as high porosity, low density, good permeability, large specific surface area, better gas transfer, more active sites in the SCR of NO_x_, and outstanding reactant shuttle space [114]. Because the above characteristics can be optimized by various methods, considerable efforts have been devoted to the development of high-efficiency and environmentally friendly denitrification catalysts, which can work well at low temperatures (<250 °C) [117,118]. However, previous studies reported that the reaction of SO_2_ and NH_3_ on the catalyst surface would deposit NH_4_HSO_4_ over the catalyst surface in the presence of oxygen. As a result, the active sites were blocked [5]. Therefore, the urgent challenges for industrial flue gas denitrification catalysts are to prevent the deactivation of active sites on the catalyst surface from the toxicity caused by SO_2_, alkali metals, as well as active metal oxide nanoparticles aggregating at high temperatures [114].

- Single-component HSMOs catalysts

**Figure 5:**
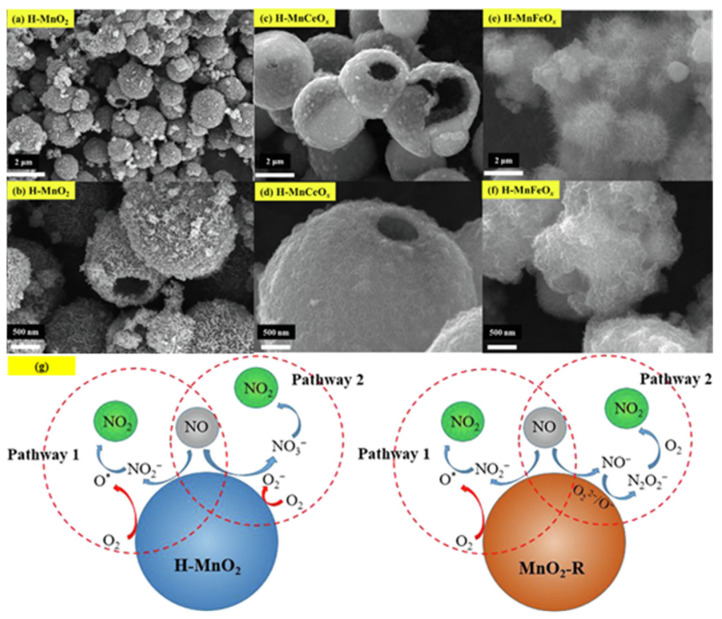
(**a**–**f**) SEM images of the MnO_x_ with hollow morphology; (**g**) Reaction pathways of NO oxidation over H-MnO_2_ and MnO_2_-R catalysts reproduced from ref. [113].

In the past decade, manganese oxide (MnO_x_) has attracted extensive attention as a low-temperature NH_3_-SCR catalyst due to its various types of active unstable oxygen. The active oxygen species is the main factor affecting its catalytic performance; meanwhile, the morphological structure also greatly affects the catalytic performance of manganese oxide (MnO_x_). For example, Shao et al. [113] reported that the hollow structure of MnO_2_ could significantly enhance the catalytic oxidation activity of NO due to the cavity structure providing the shuttle space for oxidation (Figure 5a,b). The adsorption and conversion rate of the reactants were greatly improved. As a result, the NO oxidation effect of MnO_x_ with hollow morphology was much better than that of MnO_2_-C with amorphous morphology.

The oxidation reaction pathways of NO on H-MnO_2_ and MnO_2_-R (rod-like morphology) through various characterization techniques are shown in Figure 5g. It is widely believed that the reaction pathway 1 ($\mathrm{NO}\overset{{\mathrm{Mn}}^{n+}}{\to }{{\mathrm{NO}}_{2}}^{-}\overset{O_{2}}{\to }{\mathrm{NO}}_{2}$) is the main path of NO conversion, which occurred on both H-MnO_2_ and MnO_2_-R surfaces. For H-MnO_2_, in reaction pathway 2, the NO initially converted into NO_3_^−^ on the catalyst surface. Subsequently, they were oxidized to NO_2_ by O species. However, the NO could be converted into NO^−^ and N_2_O_2_^2−^ by the abundant chemisorbed oxygen (O_2_^2−^ or O^−^ species), and then oxidized to NO_2_.

- Multi-component HSMOs catalysts

However, there are also some disadvantages of pure MnO_x_ catalysts, such as low N_2_ selectivity at high temperature, poor SO_2_ tolerance, and a narrow operation window [118]. To date, it has been reported that the use of metal dopants or promoters is a very common method to improve the SO_2_ resistance of HSMOs catalysts [112]. To improve the performance of NO oxidation, they continued to try doping the Ce and Fe into H-MnO_2_ (Figure 5c–f) and MnO_2_-R. The result indicated that the mixed-metal oxide doped with the second metal had a positive effect on the catalytic oxidation of NO. Fe doping (H-MnFeO_x_) displayed the highest NO conversion, i.e., 89.8% at 220 °C. This was because of the rambutan-like morphology of H-MnFeO_x_, which could provide more active sites.

**Figure 6:**
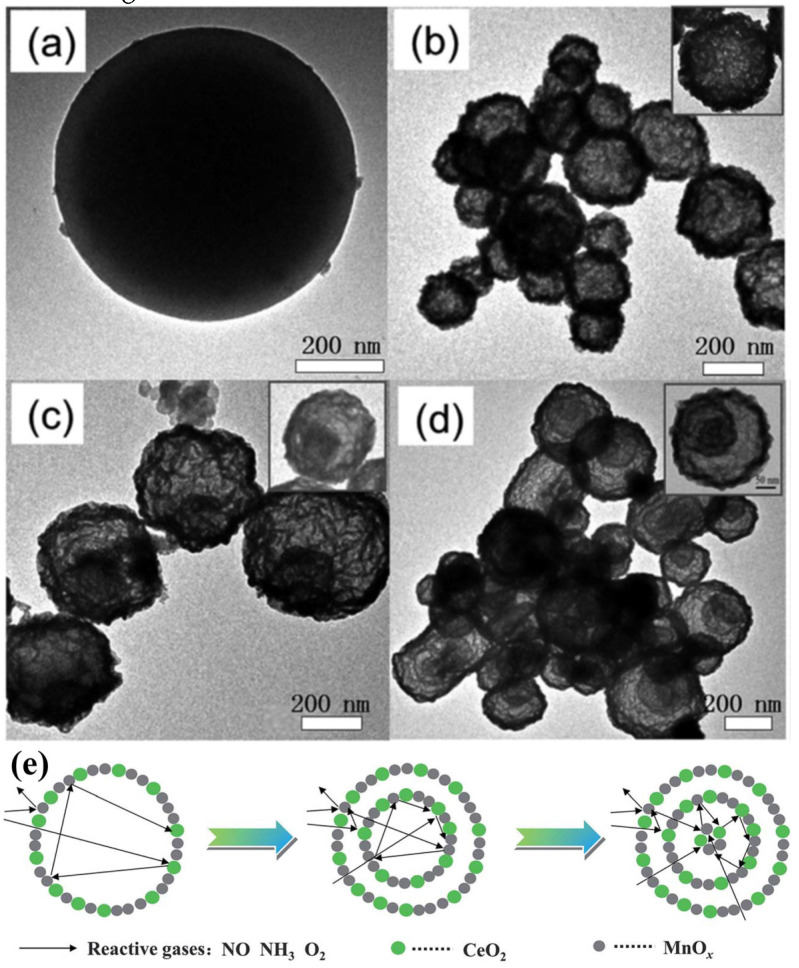
TEM images of the CeO_2_–MnO_x_ hollow spheres with various shell numbers obtained at different heating rates: (**a**) before calcination; (**b**) single-shell, 2 °C min^−1^; (**c**) double-shell, 5 °C min^−1^; (**d**) triple-shell, 10 °C min^−1^. Insets show the corresponding individual hollow sphere; (**e**) Proposed collision processes of reactive gases against hollow spheres with different shells. Figure reproduced from ref. [122].

In addition, the MnO_x_-CeO_2_ binary oxide has always been widely investigated as a catalyst of NH_3_-SCR reaction. [117]. The main reason is that the hollow structure provides a huge specific surface area, higher reducibility, and sufficient acid sites for reactants [119]. Additionally, the uniformly distributed high content of Mn^4+^ [120] and oxygen vacancies [121] were also the possible reasons. Additionally, the porous multi-shell hollow sphere can accelerate the diffusion rate of gas into the internal space. Ma et al. [122] prepared the CeO_2_–MnO_x_ composite with a multi-shell hollow structure (Figure 6a–d). The catalyst with three-layer hollow spheres presented the best performance with 100% conversion in the 150–250 °C range. As shown in Figure 6e, the multiple collisions of reactant gases between the shells were more likely to occur in the catalyst with a multi-shell structure. Therefore, the catalytic activity of the catalysts followed the order of three shells > double shells > single shell > NPs.

There are more MnO_x_ catalysts with hollow morphologies combined with other metal oxides for the NH_3_-SCR reaction, among which the typical hollow sphere morphologies were usually used. For instance, the triple-shelled NiMn_2_O_4_ hollow spheres were synthesized by a self-assembly method, which showed the low-temperature activity of the NH_3_-SCR reaction with complete NO_x_ conversion at 125 °C [123]. The excellent catalytic performance was attributed to the plentiful active Mn^4+^ and surface adsorbed oxygen of the spherical NiMn_2_O_4_ material. The hollow nanotube structure of MnCoO_x_ catalyst (MnCoO_x_-HNT) [114] was used to catalyze the low-temperature SCR process of NO_x_. The hollow nanotube structure could effectively protect the active sites on the inner surface from SO_2_ or alkali metal pollution. In addition to this, the catalyst surface possessed large amounts of OH groups, which acted as sacrificial sites for anchoring SO_2_ and alkali metals on the surface of the catalyst. In addition, another special hollow structure catalyst, such as the urchin-like MnO_x_@PrO_x_ hollow core–shell structure catalyst [124], was fabricated using a sacrificial templating method. The MnO_x_@PrO_x_ catalyst with a hollow core–shell structure exhibited excellent low-temperature NH_3_-SCR activity with a maximum NO conversion of 99% at 120 °C due to the abundant Lewis acid sites and the excellent reducibility generated by the interaction between MnO_x_ and PrO_x_. Furthermore, the special core–shell structure of the catalyst brought about the superior SO_2_ and H_2_O tolerance.

For the low-temperature NH_3_-SCR catalysts, Ce-based catalysts have also been investigated. The morphology design of the hollow structure CeO_2_ catalyst is beneficial to improve the performance of the denitration catalyst. The CeO_2_ shell can serve as the effective barrier for the aggregation of the nanoparticles. Meanwhile, the CeO_2_ shell can also improve the tolerance of SO_2_ and H_2_O by inhibiting the formation of ammonium nitrate and sulfates [125]. The CeMoO_x_ catalysts with hollow structure were investigated, such as Mo-doped CeO_2_ hollow microspheres [126] and Sn-modified CeMoO_x_ electrospun fibers [127]. The results presented that the strong redox ability, an abundance of Brønsted acid sites, plenty of chemisorbed oxygen species, and a high content of Ce^3+^ were the main factors for the excellent catalytic performance.

Thereafter, the hollow-structured CeO_2_ NH_3_-SCR catalyst, such as the hollow-structured CeO_2_-TiO_2_ catalysts [112], Cr–Ce composite catalysts with the double-shelled hollow morphology [128], CeO_2_@Fe-ZSM-5 catalyst with hollow structure [129], hollow-structured WO_3_@CeO_2_ catalyst [130] porous Ce_x_Nb_1−x_ oxide hollow nanospheres [131], and P_x_-Ce_0.3_–Zr–Ti nano-hollow spheres [132] have been developed and fabricated. The structures of these catalysts exhibited the hollow spheres. The high NH_3_-SCR activity on the hollow cavity structure catalyst was attributed to the large cavity size, increased curvature radius, abundant active oxygen species, defects, acidic strength, and increased surface proportion of Ce^3+^.

To summarize, HSMOs have been used to catalytically remove NO_x_ with NH_3_-SCR due to its unique physiochemical properties. For the catalytic oxidation activity of NO, MnO_x_ with a hollow structure has been frequently examined as a promising catalyst due to their various types of active unstable oxygen and the cavity structure could provide the shuttle space for oxidation. Additionally, transitional metals and rare earth metals doped into Mn-based oxides have been widely studied to overcome the disadvantages of MnO_x_, such as the low N_2_ selectivity at high temperature, poor SO_2_ tolerance, and narrow operation window. The exceptional catalytic activities of the Mn-based materials principally originate from the redox characteristics of MnO_x_ and the presence of oxygen vacancies. In addition, Mn-based materials and Ce-based materials with a hollow structure, which contains sufficient acid sites with large amount of active surface oxygen (O_S_), are crucial for low-temperature catalytic NH_3_-SCR. The increase in the curvature radius of the curved hollow spheres surface is an acceptable strategy.

#### 2.1.3. Catalyst for Automobile Three-Way Catalytic (TWC) Reaction and Diesel Oxidation Catalytic (DOC) Reaction

The main source of air pollution and secondary pollution is the soot particles emitted by mobile diesel engines. Therefore, it is necessary to develop high-performance catalysts that can oxidize soot at a low temperature [133]. However, both TWC and DOC should eliminate all these harmful components, such as CO, NO, hydrocarbons (HCs), and soot, at the same time [134,135]. NO_2_ is beneficial to soot combustion, as is water vapor, which is also another key factor influencing the catalytic effect of soot combustion catalysts [136].

**Figure 7:**
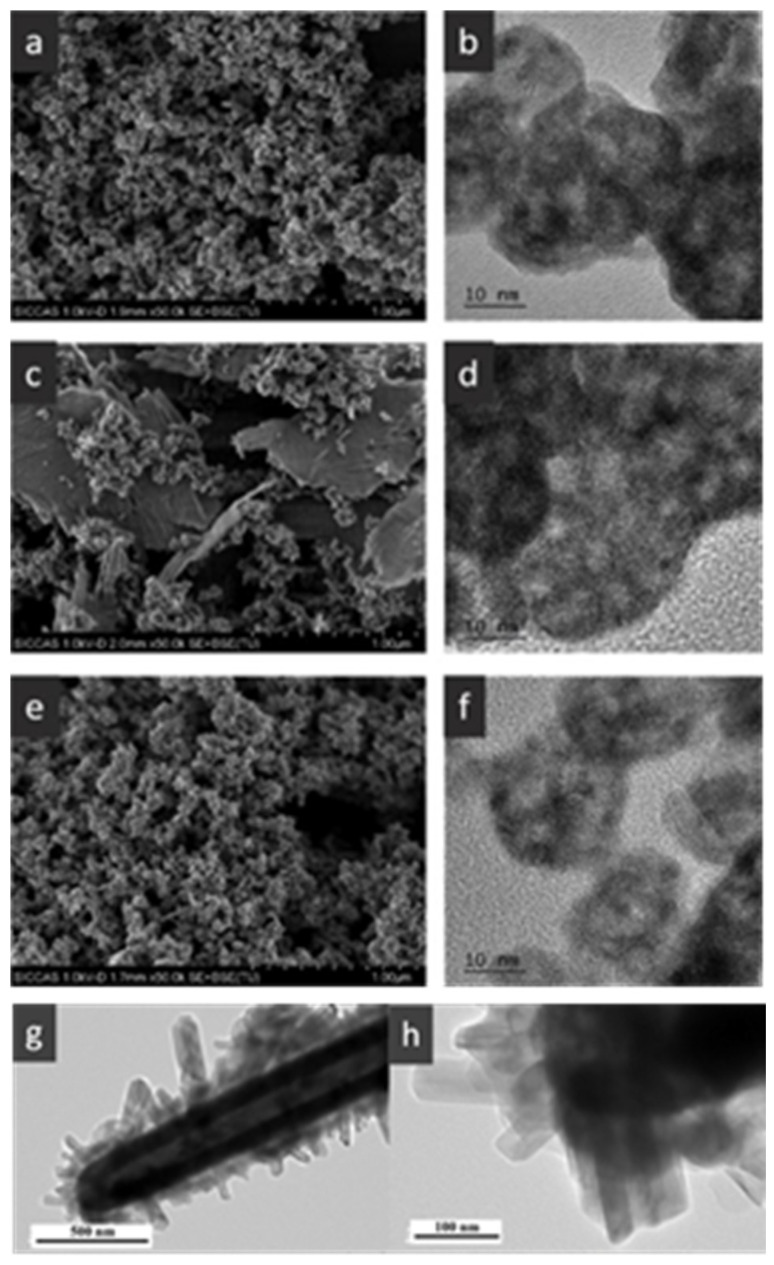
(**a**,**c**,**e**) SEM images of the CMC-Cp-a, -b , and -c, respectively; (**b**,**d**,**f**) TEM images of the CMC-Cp-a, -b, and -c, respectively (Ce/Mn/Cu molar ratio of 84/16/16 (CMC-Cp-a), 84/8/8 (CMC-Cp-b) or 84/32/16 (CMC-Cp-c)) reproduced from ref. [136]; (**g**,**h**) TEM images of the CeO_2_@MnO_2_ reproduced from ref. [139].

The hollow-structured metal oxide catalysts of the oxidation of CO and NO have reached a significantly high level of activity. The hollow structures are feasible due to the significantly improved soot–catalyst contact in the fields of automobile three-way catalysts (TWCs) and diesel oxidation catalytic (DOC) reaction [134,137,138]. Additionally, the development of the TWCs with strong interactions between metals and metal oxides to prevent the sintering of metal nanoparticles have been a research hotspot. For example, the soot or NO oxidation was performed on the CeMnCu ternary composite oxides with hollow structures prepared by different methods [136]. The results indicated that the addition of the third metal oxide, high BET surface area, small metal oxide hollow-structure grain size, uniform element distribution, and low-average-valence Ce was essential for improving the reducibility and catalytic activity of soot combustion. The hollow structure of the nanoparticles in each CMC-Cp-x (CMC: CeMnCu, Cp: co-precipitation) could expose large amounts of lattice oxygen to the (sub-) surface to promote the migration of lattice oxygen, which is very important for catalytic oxidation (Figure 7a–f).

In addition, Feng et al. [139] prepared the trepang-like hierarchical structured CeO_2_@MnO_2_ nanocomposite oxide with a width of 60 nm by hydrothermal method. The special structure of MnO_2_ short nanorods on the surface of the hollow spindle CeO_2_ (Figure 7g,h) was beneficial in terms of accelerating the oxidation of soot and achieving a high catalytic activity with T_50_ at 373 °C (5% O_2_/500 ppm NO). The main reason for this was that this unique structure could provide more active sites and increase the accessible opportunities between the catalyst and the soot.

For other hollow-structured catalysts for eliminating soot particles, La_0.63_Sr_0.2_7K_0.1_CoO_3−δ_ nanotubes with a hollow structure were synthesized by doping some Sr^2+^ to inhibit the grain growth during the heat treatment at a high temperature [140]. The soot particles have more contact chances between the catalyst and reaction gas within the hollow structure. As a result, the La_0.63_Sr_0.27_K_0.1_CoO_3−δ_ catalyst displayed high activity in terms of soot oxidation with T_50_ at 359 °C in 5% O_2_ and 2000 ppm NO. Additionally, the hierarchical hollow structure [HHS] assembled from the porous NiCo_2_O_4_ nanosheets was also attributed to abundant active oxygen species [141]. Therefore, the temperature at 50% soot conversion (T_50_) of NiCo_2_O_4_ nanosheets could be achieved as low as at 354 °C. Therefore, the application of hollow structures in the field of automobile TWC reaction and DOC reaction is valid, but still needs to be developed diligently.

### 2.2. Volatile Organic Compounds Emission Control

Volatile organic compounds (VOCs) are considered air pollutants that are greatly harmful to human health, and refer a class of substances composed of various organic compounds with a boiling point in the range of 50 °C–260 °C at room temperature. Meanwhile, outdoor sources are the main part of the anthropogenic emission sources of VOCs, including chemical industries, transportation, petroleum refineries, dry cleaners, food processors, and textile manufacturers, etc. [142]. Excess VOCs are emitted by indoors and natural resources, such as solvents and cleaning products, restaurant and domestic cooking, office supplies, printers, heat-exchanger systems, etc. [143]. Additionally, VOCs also participate in the formation of photochemical smog and the depletion of the ozone layer, which are responsible for the climate and environmental changes [144]. The catalytic recovery technologies of volatile organic compounds, such as the catalytic combustion [144], catalytic decomposition at room temperature [145], catalytic oxidation, [23], and photocatalytic mineralization, etc. [143], have been widely considered as the most promising post-treatment technologies to control the emissions of VOCs [146]. Recently, the catalytic oxidation of VOCs has received more and more attention. Therefore, it is urgent to develop excellent catalysts with advanced low-temperature activity for VOCs though structural engineering.

#### 2.2.1. Catalytic Elimination of Toluene

The aromatic hydrocarbons, such as benzene and toluene, are the toxic and carcinogenic volatile organic compounds in the discharged exhaust gas [147]. HSMOs are expected to catalyze the oxidation of these VOCs with high efficiency due to the large surface area and abundant oxygen vacancies, which are essential for the improvement of the catalytic oxidation of toluene [147,148]. Nevertheless, HSMOs have been widely used in the field of the catalytic elimination of aromatic hydrocarbons. In recent years, various excellent metal oxide catalysts with hollow structures have been developed for the catalytic elimination of toluene and have shown good low-temperature catalytic activity, stability, reusability, and excellent water tolerance [149,150,151]. However, hollow-structured metal oxides have different morphologies and compositions. Thus, the metal oxide catalysts with hollow structures for toluene oxidation reported in the summary are exhibited in Table 6. The metal oxide catalysts could be divided into single-component metal oxide catalysts with hollow structures, hollow-structured metal-oxide-supported catalysts, and hollow-structured binary metal oxide catalysts.

- Single-component metal oxide catalysts with hollow structure

The morphology of catalysts with hollow structures consisting of a single-metal oxide component, such as CeO_2_, Co_3_O_4_, and MnO_2_, has been extensively studied. For example, Feng et al. [147] prepared CeO_2_ with different morphologies (rod, cube, and hollow sphere) for catalytic toluene combustion at low temperature. Among these catalysts, the CeO_2_ hollow sphere exhibited the best tolerance to water and toluene combustion activity with T_90_ at 207 °C, which was an improvement compared to the CeO_2_ rod and CeO_2_ cube. The excellent catalytic performance of the CeO_2_ hollow sphere led to more active surface oxygen and better redox properties on the catalyst due to the larger surface area and more surface oxygen vacancies. Similarly to hollow Co_3_O_4_ polyhedral nanocages, the other reason for the high activity were the strongest OH groups and the higher atomic ratio of Co^3+^/Co^2+^ on the catalyst surface [152].

Manganese oxides (MnO_x_) are among the most active oxides for catalytic VOCs oxidation. Furthermore, a study showed that Mn was the main activity center of toluene oxidation [155]. The probable reaction pathway for the toluene oxidation of the MnO_x_ polyhedra with a hollow structure was proposed. At the start, toluene molecules were adsorbed on the surface of the catalysts, and then partly oxidized into benzyl alcohol, which might subsequently transform into benzaldehyde and benzoic acid. With the increasing temperature, the benzene ring was opened to form the maleic anhydride and was further oxidized to carbon dioxide and water [153].

**Figure 8:**
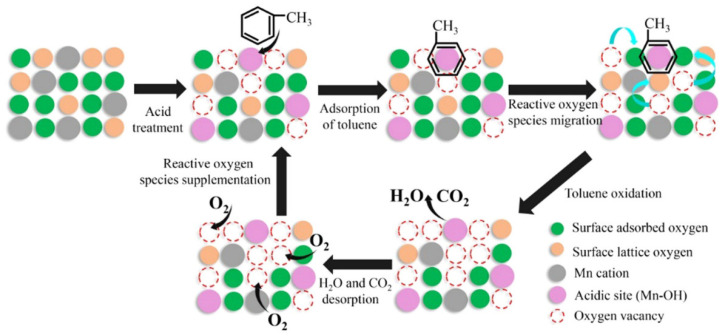
Schematic diagram of the complete reaction cycle for the catalytic oxidation of toluene on MnO_2_-1.2. Figure reproduced from ref. [151].

Moreover, the structure–activity of this important transition metal oxide has been widely studied. The VOC decomposition of MnO_x_ was ascribed to the outstanding adsorption capacity, high mobility of oxygen, the higher average oxidation state (AOS) of Mn, and abundant OH groups [159,160]. The low-temperature reducibility of the catalyst was attributed to the high content of Mn^4+^, which facilitated the occurrence of the redox cycle and promoted the activation of the surrounding surface lattice oxygen, thus enhancing the mobility of oxygen species with the participation of oxygen vacancies. Additionally, Gu et al. [151] prepared the hierarchically structured flower-like MnO_2_ hollow microspheres with low-temperature activity and high thermal stability, resulting from its large specific surface area (214 m^2^/g), abundant oxygen vacancies, improved reducibility, high number of acidic sites, and strong acidity. The adsorption and activation of gaseous toluene molecules were further promoted by these features, thus exhibiting remarkable activity for toluene catalytic oxidation at low temperature. The mechanism for explaining the results was proposed as shown in Figure 8, which was a complete cycle synergizing the Brønsted acid site and oxygen vacancy for toluene oxidation. Initially, the gas toluene was adsorbed and activated on Brønsted acid sites. Then, it reacted with the surrounding active oxygen species to produce carbon dioxide and water and complete a catalytic cycle. Meanwhile, the molecular oxygen could be activated on the oxygen vacancies, which would be generated with the consumption of active oxygen species. And, the activated surface lattice oxygen could also participate in the reaction.

**Figure 9:**
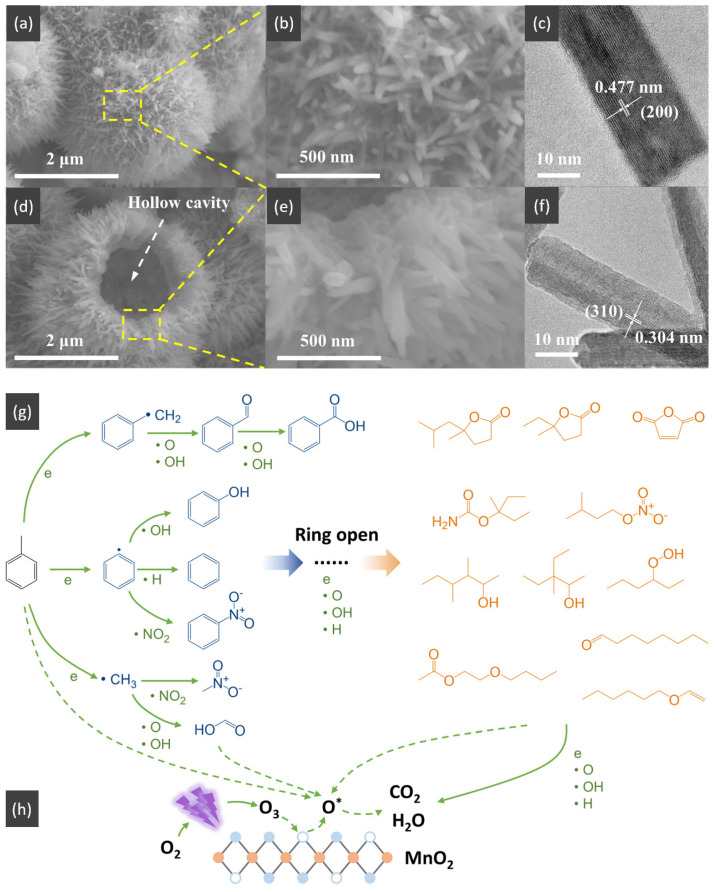
SEM and HRTEM images of: (**a**–**c**) solid-urchin and (**d**–**f**) hollow-urchin MnO_2_. Plausible reaction pathways for toluene decomposition in the PPC (post-plasma catalysis) process are also given: (**g**) NTP (non-thermal plasma)-induced gas-phase reactions in the DBD (dielectric barrier discharge) reactor and (**h**) catalytic reactions on the surface of MnO_2_ in the catalytic reactor. Figure reproduced from ref. [159].

For the oxidation elimination of VOCs, post-plasma catalysis (PPC) is also an important catalytic technology. The insufficient adsorption of gas and low catalytic activity at room temperature for the complete oxidation of toluene are still challenges in post-plasma catalysis (PPC). At the same time, hollow structures are a special morphology for metal oxides and have attracted considerable attention due to their well-defined interior voids, high specific surface areas, and superior permeation properties. Yang et al. [159] described a simple one-step template-free hydrothermal method for the preparation of the hierarchical hollow urchin structured MnO_2_, which is displayed in Figure 9a–f. The post-plasma catalytic decomposition of toluene was conducted at room temperature. As a result, hollow urchin α-MnO_2_ exhibits a higher toluene decomposition, CO_2_ selectivity, and carbon balance compared with solid urchin α-MnO_2_. The toluene decomposition, CO_2_ selectivity and carbon balance over hollow urchin α-MnO_2_ reach up to ~100%, ~59%, and ~81% at an SIE of 240 J/L, respectively, which indicated the higher activity in comparison to the non-thermal plasma (NTP) process (the initial concentration of toluene was kept at 105 ppm with a gas flow rate of 150 mL min^−1^). As shown in Figure 9g,h, the major degradation pathways of toluene over the hollow urchin α-MnO_2_ catalyst during the post-plasma catalytic process consisted of two steps: (1) the plasma-induced ring-opening destruction of toluene in the gas phase (Figure 9g); and (2) the adsorption and conversion of toluene and organic byproducts into CO_2_ and H_2_O on the surface of the catalyst (Figure 9h).

- Hollow-structured metal oxides supported catalysts

As can be observed in Table 6, the catalytic activity of single-component metal oxides is significantly lower than that of multi-component catalysts. Metal Pt and Pd noble-based catalysts have been widely investigated in the catalytic oxidation of toluene. Additionally, noble metals exhibit much higher activity in the combustion of toluene compared with transition metal oxides (mainly CoO_x_, MnO_x_, etc.), which was attributed to the advantages of a hollow structure such as large surface areas and space inside, abundant oxygen vacancies, SMSIs, etc.

To optimize the dispersion and chemical state of Pt species, Kondratowicz et al. [144] deposed Pt species into the hollow ZrO_2_ spheres as support using the bottom–up strategy and impregnation with PtCl_4_ or the reduction of PtCl_4_ in an ethylene glycol solution. In contrast with the materials produced by the bottom–up strategy, the catalyst produced by the polyol technique had a better catalytic activity in terms of toluene combustion due to the larger Pt nanoparticles with higher stabilization and the dispersion of the metallic Pt phase. Moreover, the more reactive oxygen would generate on the surface Pt site.

Mo et al. [154] prepared a series of different MnO_2_ crystal structures (α-, β-, γ-, and hollow-MnO_2_). Compared with α-, β-, and γ- MnO_2_, the H-MnO_2_ catalyst exhibited a superior high level of activity with T_90_ at 230 °C for toluene oxidation due to the abundant surface O_ads_ species on the catalyst with the well-defined hollow structure. Then, these catalysts were decorated by Pt NPs on the MnO_2_ crystal structures. The Pt/α-MnO_2_ catalyst showed the best performance for catalytic toluene combustion (T_90_ = 170 °C), which was attributed to the SMSIs between the Pt nanoparticles and the supports. The surface oxygen vacancies and the mobility of the surface lattice oxygen would be improved by the SMSIs, thus leading to the deep oxidation of the toluene molecules to CO_2_ and H_2_O.

**Figure 10:**
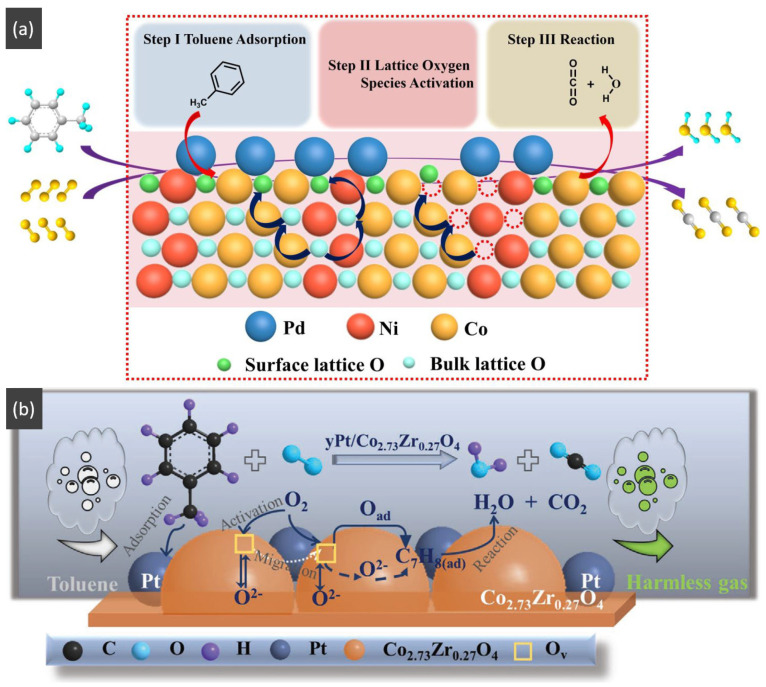
(**a**) The schematic illustration of the reaction mechanism for toluene oxidation over Pd/metal oxide catalysts reproduced from ref. [6]; (**b**) Proposed mechanism for enhanced catalytic oxidation toward toluene over 2.0 wt% Pt/Co_2.73_Zr_0.27_O_4_ reproduced from ref. [20].

Qu et al. [6] presented the h-NiCoO_x_ catalyst with large surface areas, abundant surface hydroxyl groups and numerous oxygen vacancies, which exhibited a superior catalytic activity compared with single metal oxides (Co_3_O_4_ and NiO) and NiCoO_x_ nanosheets. After being loaded with Pd particles, the 2.0 wt% Pd/h-NiCoOx demonstrated an especially high performance for toluene oxidation with approximately 100% conversion achieved at 190 °C. In comparison with h-NiCoOx, the temperature is lower by 60 °C. Mechanisms based on the Mars–van Krevelen model [161] for the toluene oxidation reaction over Pd/metal oxide catalysts are presented in Figure 10a. The proposed system was followed as: (1) after the toluene molecules were adsorbed on the catalyst surface, it was activated to form the dehydrogenated intermediates with the promotion of surface hydroxyl groups on the catalyst [162]. (2) The activated lattice oxygen species were migrated to react with the intermediates due to the existence of oxygen vacancies and mixed-valence states, such as in the Co^3+^/Co^2+^ and Ni^3+^/Ni^2+^ pairs, and then, it could be reoxidized by the gaseous oxygen [161,162]. (3) The dehydrogenated intermediates and toluene were completely oxidized from CO_2_ and H_2_O.

Wang et al. [20] reported that hollow nanocage-shaped γPt/Co_3−x_Zr_x_O_4_ catalysts show significant activity for complete toluene catalytic oxidation. The key points of the preparation with the solution-phase cation exchange method for designing this catalyst were constructing solid-solution supports by doping Zr into the Co_3_O_4_ lattice, and subsequently loading Pt. The SEM images of the materials are displayed in Table 6. After Pt nanoparticle loading, the 2.0 wt% Pt/Co_2.73_Zr_0.27_O_4_ catalyst achieved complete toluene catalytic oxidation at 180 °C, which was the best catalytic activity among these γPt/Co_2.73_Zr_0.27_O_4_ samples. In Figure 10b, which shows the proposed mechanisms of toluene decomposition and catalytic oxidation over 2.0 wt% Pt/Co_2.73_Zr_0.27_O_4_, the steps are as follows: firstly, because the Pt metal on the catalyst surface was more active, the toluene molecule favorably adsorbed onto it and was then activated as a dehydrogenation intermediate. Afterward, the major intermediates such as benzaldehyde and benzoate were formed due to the dehydrogenation intermediate reacting with active oxygen species, and finally, was completely oxidized to CO_2_ and H_2_O. Additionally, the rapid activation of O_2_ molecules benefited from the Pt metallic atoms and the active oxygen species produced by the generated oxygen vacancies.

- Hollow-structured binary metal oxide catalysts

The economic inapplicability of precious metals limits their application and development. Thus, transition metal oxides such as Co, Mn, etc., are expected to replace metal Pt and Pd due to their low cost and high availability when the outstanding catalytic activity for toluene oxidation is proven. Moreover, the addition of other metals could significantly improve the catalytic activity of toluene due to the synergistic effect [163].

Li et al. [156] prepared hollow-structured Mn–Ce binary oxides using carbon spheres as hard templates and applied for catalytic toluene combustion. The MnCe–OH catalyst exhibited the highest catalytic performance for toluene combustion with T_90_ at 237 °C in comparison with the MnCe–H and MnCe sample (obtained from acidic- or alkali-treated carbon spheres) attributed to the thinner and more porous shell, enhanced low-temperature reducibility, and moderate surface components (abundant Mn^4+^ and surface adsorbed oxygen). Furthermore, a large number of the defects with the Ce addition, surface adsorption oxygen, and the surface Mn^4+^ species of the Ce_a_MnO_x_ hollow microsphere with hierarchical structure were formed by redox co-precipitation method [157]. The catalytic performance for toluene combustion was significantly improved in terms of its high stability = and water resistance, even under the condition of 5 vol.% H_2_O of Ce_0.03_MnO_x_. The possible reaction mechanism for toluene catalytic oxidation over Mn–Ce binary oxides was offered based on in situ DRIFTS analyses. The oxidation of toluene underwent the following consecutive steps: initially, toluene molecules were transformed into aldehydic, then into benzoate species, and the CO_2_ and H_2_O were formed finally.

**Figure 11:**
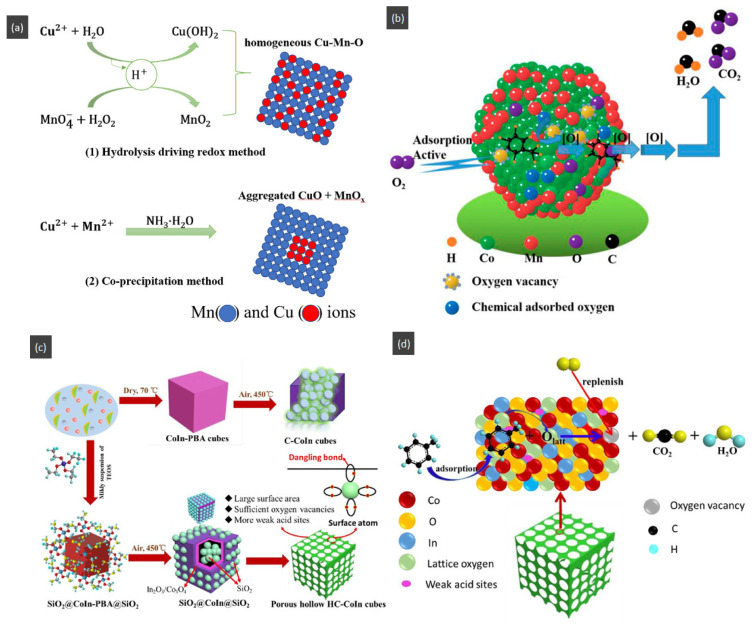
(**a**) Illustration of two pathways to synthesize the Cu–Mn oxide reproduced from ref. [155]; (**b**) Schematic of the oxidation of toluene on Mn_x_Co_3−x_O_4_ reproduced from ref. [158]; (**c**) The formation schematic of the porous hollow HC-CoInO_x_ nanocube; (**d**) The proposed reaction mechanism over the CoInO_x_ catalyst reproduced from ref. [150].

Additionally, adding a second metal element to the hollow structure to improve the catalytic activity is closely related to the preparation method. Xiao et al. [155] reported the hollow-microsphere CuMnO_x_ catalysts synthesized by an expeditious salt hydrolysis-driven redox-precipitation protocol for toluene combustions. As shown in Figure 11a, to fabricate the CuMnO_x_ with molecular-scale homogeneity and a high dispersion of Cu^2+^ and Mn^2+^, the hydrolysis driving redox method was used to raise their atomic utilization efficiency compared with the co-precipitation method. The HR-2Mn1Cu (hydrolysis-driven redox-precipitation protocol) showed the lowest toluene conversion temperature with T_50_ and T_90_ of 228 °C and 237 °C, respectively, and the toluene conversion at 240 °C was much higher than that of other catalysts. The excellent activity of the HR-2Mn1Cu was ascribed to the formation of a long-range disordered mesostructure with the uniform introduction of copper ions by the hydrolysis-driven redox co-precipitation. With the corrosion of H_2_O_2_, the surface hollow structure and accumulative pores were formed, which then increased the high specific surface area and accessibility of surface edge sites and inner atoms of HR sample.

Temperatures and heating rates are important heating decomposition conditions for precursors, based on the application of hollow Co_3_O_4_ polyhedral nanocages [152]. Zhao et al. [154] successfully fabricated the hollow Mn_x_Co_3−x_O_4_ polyhedron (HW-Mn_x_Co_3−x_O_4_) by controlling heating conditions to optimize the decomposition of Mn@Co-ZIFs precursors. The HW-Mn_x_Co_3−x_O_4_ displayed remarkable catalytic oxidation performance for toluene with T_100_ occurring at 195 °C due to the high surface atomic ratio of Co^2+^/(Co^3+^ + Co^2+^), an abundance of surface-adsorbed oxygen with the largest specific area, and a minimum crystallite size. In addition, the possible reaction mechanism was proposed and followed the L-H mechanism different from hollow Co_3_O_4_ polyhedral nanocages. The complete redox cycle [164] is shown in Figure 11b, and the steps were as follows: firstly, the toluene molecule reacted with the chemically adsorbed oxygen after being adsorbed onto the surface of the catalyst. Secondly, the benzaldehydic species were produced and converted into CO_2_ and finally H_2_O. Meanwhile, oxygen vacancies were produced by the catalysts to form the new chemically adsorbed oxygen.

The hollow-structured material made good progress, but the synthesis of the hollow-structured cubic metal oxides faces huge challenges. As shown in Figure 11c, the SiO_2_ template strategy was applied to prepare hollow the CoInO_x_ nanocube (HC-CoInO_x_) [150] for the catalytic combustion of toluene. The formation process of the construction of a hollow structure indicated that using the porous SiO_2_ template can greatly increase its surface area to produce a large number of surface dangling bonds and provide more oxygen vacancies and surface weak acid sites, which would play important roles in improving the oxidation activity of materials. Thus, the hollow HC-CoInO_x_ nanocube exhibited an excellent catalytic performance (T_90_ = 178 °C). The proposed reaction mechanism over the hollow HC-CoInOx catalyst for the toluene oxidation process is shown in Figure 11d, which preferred the Mars–van Krevelen mechanism. The redox cycle includes the following steps: firstly, toluene molecules reacted with the adjacent lattice oxygen after adsorbing onto the metal active sites and then formed CO_2_, H_2_O, and an oxygen vacancy. Subsequently, the gas O_2_ molecules were reabsorbed and replenished into this oxygen vacancy, and then reacted with another toluene molecular as before.

In summary, there is no doubt that the optimization of the hollow structure highly improved the catalytic activity of single-component metal oxide catalysts. However, there are bottlenecks to this improvement. The introduction of noble metals is known to further enhance the activity. For the supported catalyst, the precious metal was the active site to activate the O_2_ molecules, and the active oxygen species were produced by the generated oxygen vacancies of the HSMOs [20]. Additionally, to make full use of the advantages of the hollow structure (high specific surface areas, big pore volumes, abundant defects, adjustable morphology, space utilization, low density, and the metal–support interactions, etc.) [159], the toluene catalytic oxidation reaction is crucial.

In order to maximize the toluene catalytic oxidation activity of the HSMOs, the incorporation of other active metal elements has been used to adjust the structure–activity relationship. The key factors for the oxidation of toluene on the catalyst are the surface reaction [158], the high number of acidic sites [151], and the supply of active oxygen by the abundant oxygen vacancies [150]. As for binary metal oxide catalysts, the catalytic oxidation of toluene over the metal oxides with a hollow structure is related to various influencing factors, such as the concentration of surface oxygen vacancies [20], the thickness and the porosity of the shell [156], abundant surface hydroxyl groups [6] and Mn^4+^ species [157], and so on. Based on these synthesis methods and experimental investigations, the hollow-structured metal oxide catalysts with high activity and durability at the component level could be designed and fabricated.

#### 2.2.2. Removal of Other Volatile Organic Compounds (VOCs)

Formaldehyde (HCHO) has been considered a carcinogenic and toxic volatile organic compound (VOC), which widely exists in wood adhesives, furniture, preservatives and disinfectants, textiles, dyes, cigarette smoke, and other materials we encounter daily [23]. As a dangerous indoor pollutant, HCHO at a very low concentration can also pose a huge threat to human health [165]. In addition, chlorinated volatile organic compound pollutants (CVOCs) also include chlorinated volatile organic compounds which have high chemical stability, severe toxicity, and potential carcinogenicity [166]. The removal of pollutants in the air or indoors using catalytic oxidation technology has been considered one of the most promising technologies for addressing this issue.

Compared with traditional catalytic materials, HSMOs have attracted significant attention for addressing catalytic VOC oxidation due to certain advantages such as their large specific surface area, low density, high loading capacity, outstanding interior voids, good surface permeability, excellent permeation properties, and high mobility. Furthermore, HSMOs are good candidates for catalyst support.

The related catalytic elimination method, catalytic performance, synthesis method, and structural properties of the previously reported metal oxide catalysts with hollow structures in catalytic oxidation are summarized in Table 7. As can be observed, the application range of hollow-structured oxide catalysts in the field of VOC removal is gradually expanding due to their outstanding advantages. The catalysts were prepared by different methods and metal oxides led to the exhibition of various morphologies, such as hollow spherical structures, hollow nanoboxes, hollow chains, and core–shell nanospindles.

- Hollow nanospheres

For vinyl chloride (VC) catalytic oxidation, Wang et al. [166] synthesized hollow alumina microspheres (Al_2_O_3_-hms) as the support for ruthenium/cobalt binary oxides to prepare the catalyst. The RuCoO_x_/Al_2_O_3_-hms exhibited the highest VC oxidation activity with T_50_ at 310 °C and T_90_ at 345 °C compared with RuO_x_/Al_2_O_3_-hms, CoO_x_/Al_2_O_3_-hms, and Al_2_O_3_-hms. The SMSI effects between metal nanoparticles and Al_2_O_3_-hms support varied the low-temperature reducibility, the abundance of surface oxygen, lattice oxygen mobility, and the metal valence state distribution over RuCoO_x_/Al_2_O_3_-hms catalysts.

Manganese oxides (MnO_x_) were the transition metal oxides with multiple crystalline phases and oxidation states, which have been extensively investigated due to their high reductive degree in high oxidation states (Mn^4+^ and/or Mn^3+^). Chen et al. [167] synthesized manganese oxide honeycomb and hollow nanospheres via a simple soft chemistry route for the effective removal of HCHO. The catalytic activity of K_x_MnO_2_ nanospheres for HCHO oxidation was improved by changing the KMnO_4_/OA molar ratio to form the hollow structure. The 100% HCHO conversion temperature (T_100_) of the hollow K_x_MnO_2_ nanospheres was 80 °C, and the T_100_ of honeycomb nanospheres was 85 °C, which, due to the HCHO, would adsorb and retain for a longer period in the hollow structure.

**Figure 12:**
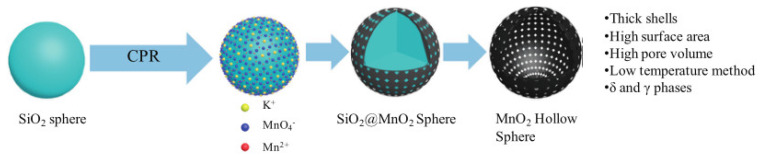
Brief description of the hard templating method to synthesize the MnO_2_ hollow sphere. Figure reproduced from ref. [23].

Boyjoo et al. [23] prepared the MnO_2_ hollow spheres by the redox (CPR) method to control the manganese oxide precipitation coated on SiO_2_ spheres (Figure 12). The Mn[P]N (‘Mn’ stands for MnO_2_, ‘P’ for permanganate Mn(VII) solution, and ‘N’ for nitrate Mn(II) solution; the letter between the square brackets [] represents the solution that was added dropwise) was performed best with T_50_ and T_90_ of 75.6 °C and 99.7 °C, respectively, which was attributed to the highest surface area. The Mn[P][N] maintained a high 75% conversion up to 90 h of reaction due to the high concentration of oxygen vacancies to continuously regenerate hydroxyl species on the surface of the birnessite sheets. Moreover, the hydroxyl radicals replenished from the water would be helped to complete the oxidation of formaldehyde.

Furthermore, the adoption of a hollow spherical structure with hierarchical structures to modify the surface of metal oxides has received more attention due to open the hierarchical architecture endowed abundant surface sites for the diffusion and adsorption of reactants as well as the high active mental dispersion [2]. For example [168], MnO_2_ hierarchical hollow microspheres (MnO_2_-S3) with a crystalline structure of γ-MnO_2_ were prepared using the hydrothermal method; then, the Au nanoparticles were dispersed on the surfaces of hollow MnO_2_ microspheres using the sol–gel method. The Au/MnO_2_-S3 showed the highest activity with 59.2% conversion, achieved at 25 °C, among the Au/MnO_2_ samples. Therefore, another effective method was to load noble metals to improve the catalytic activity.

**Figure 13:**
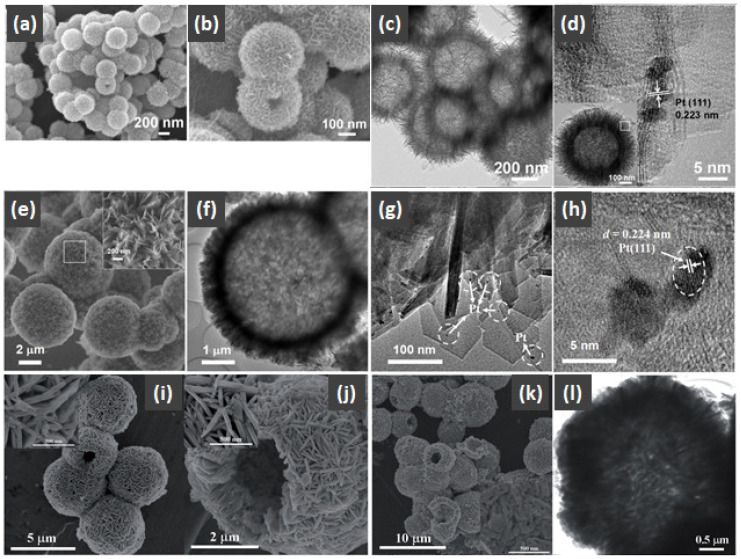
FESEM images of the MnO_2_-HCS (**a**,**b**). TEM ((**c**) and inset of (**d**)) and HRTEM (**d**) images of the Pt/MnO_2_-HCS reproduced from ref. [2]; (**e**–**h**) Structural characterization of the Pt/γ-Al_2_O_3_; SEM (**e**), high magnification SEM (inset in (**e**)), TEM (**f**,**g**), and HRTEM (**h**) images of the PHAO sample reproduced from ref. [169]; (**i**–**l**) SEM images and the corresponding high-magnification SEM images (insets) of the samples: Ni80 (**i**), Ni400 (**j**), and Ni600 (**k**); TEM image of the Ni400P sample (**l**) reproduced from ref. [170].

Pt with high activity and good stability is typically used to decompose HCHO, even at room temperatures [173]. This was attributed to the fact that the metal Pt with the negative charge could provide more active sites for HCHO oxidation, probably due to facilitating the electron transfer and the formation of active oxygen [174]. The HCHO conversion efficiency of the hierarchical WO_3_ nanoflakes, comprising assembled hollow microspheres, was only 3% within 60 min, and the HCHO conversion efficiency was 97% of WO_3_-Pt1.0 (within 60 min) (1.0 represents the weight percentage of Pt loaded in the samples) [165]. This result indicates that WO_3_ was inert for the catalytic oxidation of HCHO, and Pt played a key role in the HCHO decomposition. Furthermore, Sun et al. [2] prepared a hierarchical core–shell Pt/MnO_2_-HCS (hollow carbon spheres) composite sphere through the template-assisted hydrothermal method and reductive deposition of Pt NPs (Figure 13a–d). Compared with MnO_2_-MS (MnO_2_ microsphere), the Pt/MnO_2_-HCS sample exhibited a higher HCHO decomposition efficiency of 90.5% within 60 min.

The other special hierarchical macro-mesoporous hollow structure, such as that of the γ-Al_2_O_3_ hollow spheres (HAO), was assembled with nanosheets on the surface and then PHAO (Pt- hollow γ-Al_2_O_3_ spheres) was fabricated by depositing small Pt NPs on the surfaces of the nanosheets, which were used for HCHO oxidation (Figure 13e–h) [169]. Among all the catalysts, PHAO was the most active catalyst in terms of HCHO oxidation and demonstrated a highly improved performance.

As shown in Figure 13i–l, Qi et al. [170] prepared hierarchical Pt/NiO hollow microspheres through the template-free approach, and loaded Pt by the combined NaOH-assisted impregnation of NiO with NaBH_4_ reduction. In contrast to Ni400G (the as-prepared sample heated to 400 °C which was ground into a fine powder using an agate mortar to destroy the hollow spheres), the Ni400P (with a calcination temperature at 400 °C) achieved a higher catalytic activity due to the hierarchical hollow spheres assembled by a large number of nanosheets and the bimodal macro-mesoporous structures. And, this phenomenon also reflected the two following points: (1) the diffusion and transport of gas molecules would be blocked in the pores generated by the disorderly stacked nanosheets; (2) it is difficult for the growth and cluster of Pt nanoparticles because of the lack of a hierarchical macro-mesoporous structure.

- Other hollow-structured metal oxides

To oxidate HCHO, Qi et al. [145] prepared a highly efficient Pt/TiO_2_ catalyst using TiO_2_ hollow chains as support material, and found that the hollow chain-like structure with numerous mesopores, great pore volume, and a high surface area remarkably improved its catalytic activity. Additionally, the uniform CoSn(OH)_6_ hollow nanoboxes with an abundance of surface hydroxyl groups and plenty of catalytic oxidation sites also provided superior support in order to disperse the Pt metals [171].

**Figure 14:**
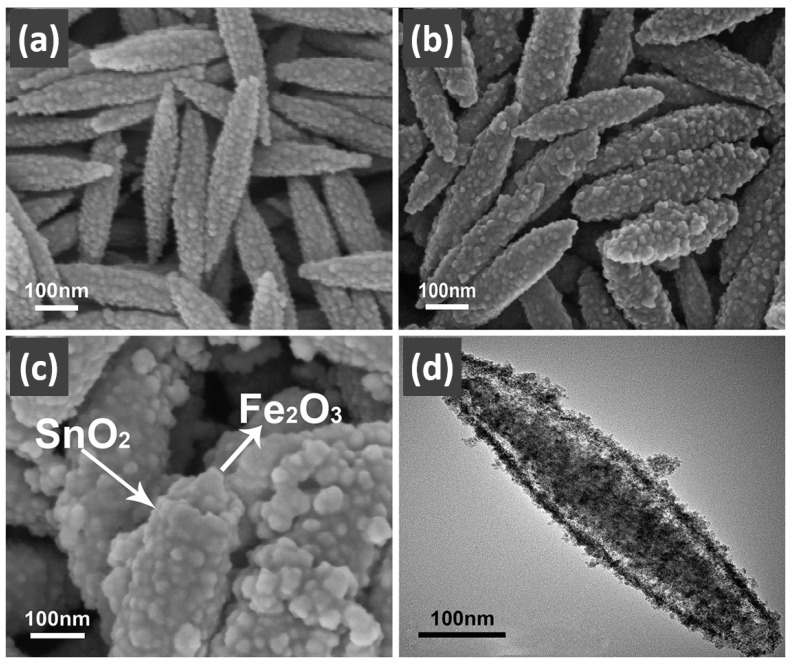
The SEM and TEM images of the prepared Fe_2_O_3_ (**a**) and Fe_2_O_3_@SnO_2_ (**b**–**d**) reproduced from ref. [172].

Lv et al. [172] reported using the Fe_2_O_3_@SnO_2_ core–shell nanospindles as the support for loading Pt nanoparticles, which were prepared by assembling SnO_2_ shells over Fe_2_O_3_ cores (Figure 14a–d). Compared to the Pt/Fe_2_O_3_ and Pt/SnO_2_ catalysts, the Pt/Fe_2_O_3_@SnO_2_ shows enhanced room-temperature HCHO oxidation activity with the 95.99% removal ratios of HCHO after 1 h. By analyzing the DFT simulation results, an excellent catalytic performance was ascribed to the fast adsorption and activation of the reactant O_2_ by the Fe_2_O_3_ surface, and the SnO_2_ surface is beneficial for the desorption of the resultant H_2_O. Also, the synergetic combination of the individual oxide building blocks produced by the hetero-structure was another reason for the observed enhanced catalytic capability.

Generally, metal supports loaded with noble metal are widely used catalysts for the catalytic oxidation of volatile organic compounds. However, the dispersion degree of noble metal on metal oxides is critical to catalytic activity. Metal oxides with a hollow structure have shown their advantages, such as their large specific surface area, low density, high loading capacity, outstanding interior voids, good surface permeability, and high mobility, etc. The low-temperature oxidation performance of catalysts for the removal of volatile organic compounds is due to the two following key factors: (1) the synergistic effect between the noble metal particles and the support; and (2) the abundance of surface sites for the diffusion and adsorption of reactants.

### 2.3. Removal of Other Pollutants

#### 2.3.1. Catalytic Conversion of CO_2_

As it is well known, global warming is caused by massive emissions of greenhouse gases [175]. Carbon dioxide has been considered the main greenhouse gas responsible for global warming [176,177]. Meanwhile, CO_2_ can also be a promising and economical carbon source for synthesizing organic compounds [178,179]. Thus, the incorporation of CO_2_ into epoxides [175], the synthesis of symmetrical or asymmetrical urea compounds [180] and formic acid and its derivatives [181] from CO_2_, the synthesis of acetic acid via methanol hydrocarboxylation with CO_2_ and H_2_ [182], as well as the recycling of CO_2_ through the hydrogenation or reforming processes, etc. [183], are considered promising approaches. In some of these processes, catalysts have been extensively researched and developed because they can reduce processing costs. Researchers are keen to enhance the catalytic activity using metal oxides with special surface characteristics [175]. Moreover, the hollow nano-microstructures of metal oxide materials strongly affects their efficiency due to the composition and size of such structures, the various pore sizes, and the fine structure of the spherical shells. Thus, the application of HSMOs is one of the solutions for catalysts to achieve a high performance.

**Figure 15:**
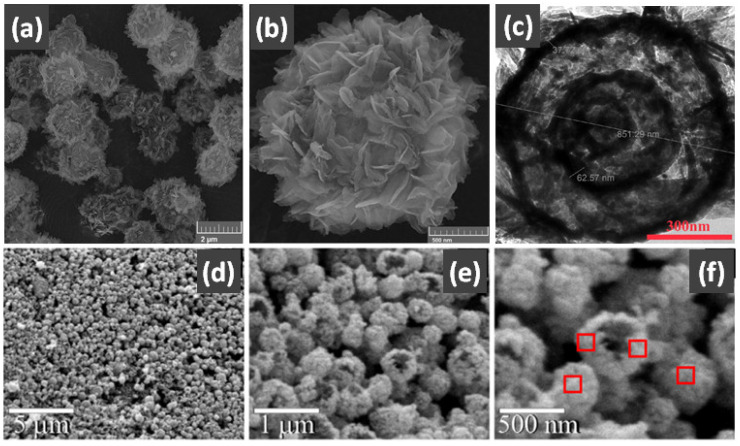
(**a**,**b**) FESEM and (**c**) TEM images of the synthesized CuAl_2_O_4_ hollow sphere reproduced from ref. [179]; (**d**–**f**) SEM images at different magnifications of the CuO-ZnO catalyst reproduced from ref. [183]. The red borders in the figure represent irregular spherical shape of the sample.

Spinel-type composite metal oxides have been widely used for the incorporation of CO_2_ and CS_2_ into epoxides due to their various advantages, such as their mixed-metal oxidation state, chemical stability, excellent synergistic performance, economical cost, and simple preparation process [9,175,176]. In terms of ingredients, catalysts with nanocrystalline aluminum-derived spinel structures are widely employed due to their high thermal stability, high mechanical resistance, hydrophobicity, and low surface acidity. The hollow spinel-type composite metal oxides, such as nano-CuAl_2_O_4_ hollow spheres [175], nanoporous triple-shelled CuAl_2_O_4_ hollow spheres [179], and multi-shell hollow CoAl_2_O_4_ microspheres [9], have been promising catalysts for the cycloaddition of CO_2_ to epoxides. For instance, the copper–alumina spinel hollow sphere decorated the unique structures of the nanoflake with a triple-shell structure [179], as displayed in Figure 15a–c, which highly improved the catalytic activity for the cycloaddition of CO_2_ at atmospheric pressure due to the good accessibility of interior active sites in the hollow structure. Additionally, another hollow-structured catalyst with an excellent performance for the cycloaddition of CO_2_ and epoxide under solvent-free conditions is that of hollow marigold CuCo_2_O_4_ spinel microspheres [176], which have numerous Lewis acidic active sites.

In addition, Witoon et al. [183] prepared a series of CuO–ZnO catalysts for the hydrogenation of CO_2_ to methanol by adjusting the chitosan concentration. It was found that the hollow-structured CuO-ZnO catalyst (Figure 15d–f) with the largest surface area (46.2 m^2^ g^−1^) and the smallest crystallite sizes achieved the highest space–time yield of methanol (135 g kg^−1^_cat_·h^−1^) at 513 K. Tian et al. [184] synthesized the hollow CuO/ZnO/Al_2_O_3_ composite microspheres using carbonaceous saccharide as the template. The obtained catalysts could achieve an optimum methanol yield of 15.3% with the 24.7% CO_2_ conversion at 262 °C.

Other hollow structures were developed from the MOF derivative catalysts. The included hollow-structured Cu@ZrO_2_ prevent the sintering of Cu nanoparticles, which leads to the high performance of CO_2_ hydrogenation, and to methanol reaction with 5% CO_2_ conversion and 85% methanol selectivity at 220 °C [185]. Additionally, the hollow-structured In_2_O_3_@ZrO_2_ effectively improved the catalytic activity of formate intermediates to methanol (STY_MeOH_ of 0.29 g_MeOH_·g_cat_^−1^·h^−1^ at 290 °C) because of the strong In_2_O_3_–ZrO_2_ interaction at the In_2_O_3_/ZrO_2_ heterointerfaces [186].

#### 2.3.2. Catalytic Conversion of CH_4_

The impact of CH_4_, another greenhouse gas, on the environment is 20 times that of CO_2_ [187]. The methane dry reforming process (Equation (3)) is a sustainable means of reducing greenhouse gas emissions by simultaneously consuming two kinds of greenhouse gases. It can contribute to both environmental protection and the energy economy [188]:

CH_4_ + CO_2_ → 2H_2_ + 2CO, △H = 247 kJ/mol (3)

However, the carbon deposition [189] and active metals sintering [188,190] of catalysts are the main problems arising during the dry reforming of methane (DRM). Recent studies have shown that the application of HSMOs is favorable for the catalysis of DRM [189,191,192,193].

As it is known to all, HSMOs exhibit advantages including their high loading capacity, superior pore permeability, high specific surface area, abundant inner void space, and low density. Therefore, the promotion of gas diffusion and the high dispersity of active metal nanoparticles were attributed to the hollow structure [191,194]. Additionally, the high metal dispersion over a greater surface area of the support is one particular advantage of supported metal catalysts, showing excellent activity due to the interactions of the metal with the support and the stability of the support at high temperature.

Compared with the one-component system, the incorporation of the second oxide significantly improves the catalytic activity, stability, and anti-catalytic toxicity of the nanostructure, which is due to the synergistic interaction between the two different oxides that would be increased by tuning the composition and morphology of hollow structure. The adjustment of the component benefited from the SMSIs provided sufficient reaction active sites and oxygen vacancies to inhibit the high carbon deposition [195]. Finally, superior catalytic activity and particle sintering resistance would be achieved. Thus, the investigation of HSMOs could remarkably influence the activity, stability, and anti-catalytic toxicity of catalysts for DRM.

#### 2.3.3. Removal of Organic Compounds

To date, HSMOs have shown great potential in the field of the catalytic environmental contaminants, such as the hydrogenation of 4-nitrophenol (4-NP) and the catalytic oxidation of 1,2-dichlorobenzene (*o*-DCB). For the catalytic hydrogenation of 4-NP, Au/CeO_2_ catalyst [196], hollow Cu_2_O/rGO nanohybrid [197], Au@mesoporous SnO_2_ yolk–shell nanoparticles [198], cellulose nanocrystal-supported hollow CuFe_2_O_4_ nanoparticles (CuFe_2_O_4_/CNC) [199], and Fe_3_O_4_@Au hollow spheres, etc. [200], the materials exhibited excellent catalytic activity due to the unique porous structure, were permeable for chemical species, and had large specific surface areas, which induced the easy accessibility of the reactant at the active sites. Furthermore, the good reusability of Fe_3_O_4_@Au hollow spheres was attributed to its magnetic properties, which resulted in the rapid recycling of catalysts [200].

**Figure 16:**
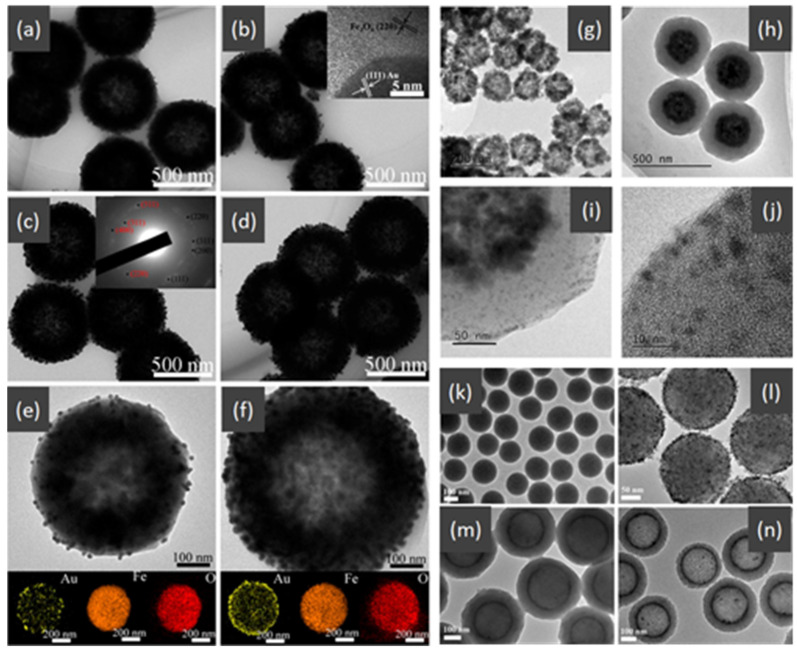
TEM images of the Fe_3_O_4_–Au 5 mL (**a**) and Fe_3_O_4_–Au 20 mL with the HRTEM image (inset) (**b**); Fe_3_O_4_–Au 40 mL with the SAED pattern (inset) (**c**) and Fe_3_O_4_–Au 60 mL (**d**); TEM images of single Fe_3_O_4_–Au 5 mL (**e**) and Fe_3_O_4_–Au 60 mL (**f**) microspheres and corresponding EDS elemental mapping images (Au, Fe, and O). Figure reproduced from ref. [201] TEM images of (**g**) hollow Fe_3_O_4_ microspheres, (**h**) Fe_3_O_4_/P(GMA-EGDMA) microspheres, and (**i**,**j**) Fe_3_O_4_/P (GMA-EGDMA)SO_3_H/Au-PPy microspheres. Figure reproduced from ref. [202]. TEM images of (**k**) SiO_2_ nanospheres, (**l**) Au/Fe_3_O_4_/SiO_2_, (**m**) Au/Fe_3_O_4_/SiO_2_@TiO_2_, (**n**) Au/Fe_3_O_4_@hTiO_2_. Figure reproduced from ref. [203].

Except for the Fe_3_O_4_@Au hollow spheres sample, hollow Fe_3_O_4_-Au nanocomposites [201] (Figure 16a–f), hollow Fe_3_O_4_/P(GMA-EGDMA)SO_3_H/Au-PPy catalyst [202] (Figure 16g–j), Au/Fe_3_O_4_@TiO_2_ hollow nanospheres [203] (Figure 16k–n), and multi-shelled FeCo_2_O_4_ hollow porous microspheres/cotton cellulose fibers (CCFs) [204] all exhibited magnetism, and thus their convenient separability and excellent repeatability. Moreover, the superior catalytic performance of FeCo_2_O_4_ hollow porous microspheres/CCFs was also ascribed to the synergistic effect between magnetic TS-FeCo_2_O_4_ (triple-shelled) and CCFs. And, the CeO_2_@Au@CeO_2_–MnO_2_ catalyst with the sandwich hollow structure [103] proved the synergistic effect among the components. Additionally, the TS-FeCo_2_O_4_/CCF materials could act as a photo-catalyst with high catalytic activity, which was attributed to the multiple additional reflections of the incident light which would occur in the multi-shelled hollow structure. Thus, the more efficient utilization of incident light within the interior cavity was realized.

Furthermore, the Co_3_O_4_/CoP composite hollow polyhedron [7] was synthesized as a superior catalyst with dramatic efficiency and stability for the reduction of 4-NP through phosphorization calcination. It is believed that the rapid transfer of electrons during the catalytic hydrogenation of 4-NP is crucial, which is supported by the MFe_2_O_4_ (M=Co, Ni, Cu) hollow spheres [205], the plasmonic Au-loaded hollow porous TiO_2_ spheres, etc. [206].

**Figure 17:**
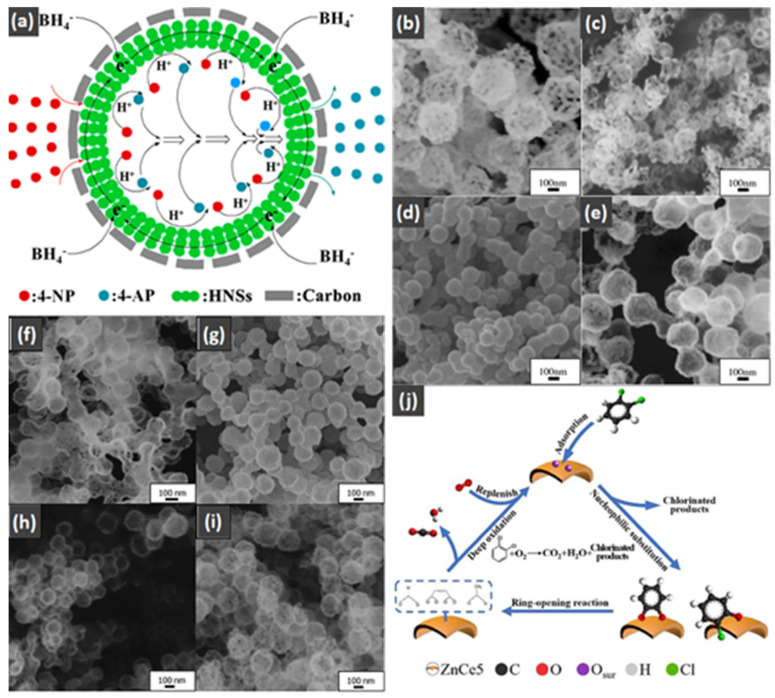
(**a**) Schematic diagram of the catalytic mechanism of metal oxide HNSs@C for the hydrogenation of 4-NP reaction. Figure reproduced from ref. [207]. SEM images of (**b**) Fe_2_O_3_, (**c**) FeCa5, (**d**) FeCa10, and (**e**) FeCa20. Figure reproduced from ref. [209]. SEM images of (**f**) FeMn10, (**g**) FeMn20, (**h**) FeMn40, and (**i**) FeMn80 (represent the molar ratios of Mn/(Fe + Mn) are ~10, 20, 40, and 80 mol%). Figure reproduced from ref. [22]. (**j**) Proposed reaction routes of *o*-DCB catalytic oxidation over ZnCe5 (doped with 5 mol% Ce). Figure reproduced from ref. [210].

In addition, Wu et al. [207] prepared metal oxides (NiO, CuO, and NiO/CuO) with hollow nanosphere morphology. The five-stage hydrogenation of the 4-NP reaction process over NiO/CuO porous carbon shell (HNSs@C) is illustrated in Figure 17a. The HNSs played a key role in the “electrical” connection between the particles during the electron transfer from the oxidation site to the reduction site [208]. Moreover, the surface with rich interconnected nanobranches could reduce the interface resistance and provide a convenient way for electron transfer.

For the catalytic oxidation of *o*-DCB, the structure–reactivity relationship has been widely investigated. However, the morphological effect of the hollow structure was rarely discussed. A small amount of data currently available developed demonstrated that the strong performance of the hollow-structured catalysts was attributed to their small crystallite size, the high concentration of surface-active oxygen [22], good low-temperature reducibility, and the synergistic effect between metal oxides [209,210]. As shown in Figure 17b–e, the Ca-doped FeO_x_ hollow microspheres were fabricated using carbon microspheres as templates [209], and the optimal FeCa10 (the nominal content of Ca was 10 mol%) hollow microspheres achieved a superior catalytic activity, water resistance, stability, and CO_2_ selectivity. The hierarchical porous structure of a series of novel Fe–Mn composite oxides with hollow microsphere morphology, as shown in Figure 17f–i, was also one of the properties that resulted in high catalytic activity, CO_2_ selectivity, good water-resistant performance, and excellent stability [22].

The oxidation of *o*-DCB over mixed-oxide hollow microspheres occurs to follow the Mars–van Krevelen-like mechanism [209,210]. For example, as shown in Figure 17j, the reaction mechanism of the Ce-doped ZnO hollow microspheres was summarized as the following steps: (1) *o*-DCB was converted into catecholate or phenolate species through nucleophilic substitution after being adsorbed onto the surface ZnCe5; (2) and then ZnCe5 proceeded to inducing the ring opening reaction of catecholate or phenolate species, which facilitated the formation of maleate, acetate, and formate species; (3) CO_2_ and H_2_O were formed due to the oxidation of a large amount of maleate, acetate, or formate species [210].

HSMOs also have great potential for treating water pollutants such as dyes (e.g., acid orange 7(AO7) [211], methylene blue) [212], pharmaceuticals (e.g., acetaminophen [213], norfloxacin (NOR) [214], tetracycline (TC) [215], and ciprofloxacin) [216], and organic pollutions (e.g., phenol) [217] through the photocatalytic degradation and advanced oxidation processes (AOPs).

**Figure 18:**
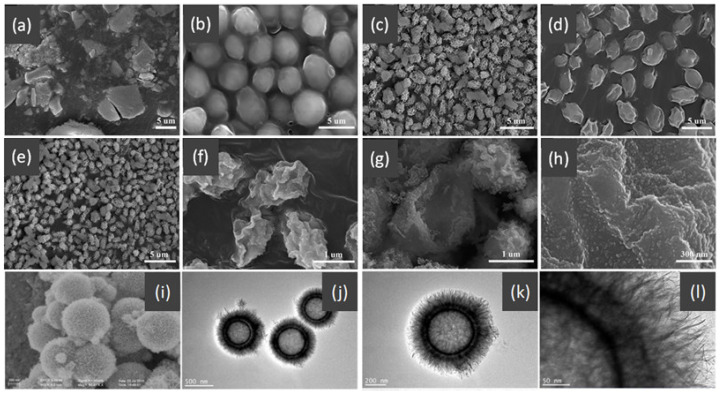
SEM images of the (**a**) Fe-doped CeO_2_ nanoparticles, (**b**) yeast template, (**c**) CeO_2_ hollow microspheres, and Fe-doped CeO_2_ hollow microspheres before (**d**) and after (**e**–**h**) calcination. Figure reproduced from ref. [211]. The SEM image (**i**) and TEM images (**j**–**l**) of Fe_3_O_4_@MnO_2_ BBHs. Figure reproduced from ref. [212].

For the photocatalytic degradation of pharmaceuticals and organic pollutions, hollow-structured TiO_2_ has been widely studied. The hollow mesoporous TiO_2_ microspheres were synthesized for the photocatalytic degradation of acetaminophen [213], and the TiO_2_ hollow mesoporous nanostructures photocatalytic degradation of phenols [217]. Additionally, the CeO_2_/Co_3_O_4_ hollow microsphere [215] was prepared for the degradation of tetracycline (TC). For the photodegradation of AO7, Fe-doped CeO_2_ hollow microspheres [211] as the catalyst were prepared by a simple coprecipitation method using yeast as a bio-template, as shown in Figure 18a–h. Fe-doped CeO_2_ hollow microspheres have a higher photocatalytic performance in degrading AO7 aqueous solutions containing H_2_O_2_ under visible irradiation, which can be attributed to their greater number of oxygen vacancies, higher specific surface area, and lower band gap, in contrast with CeO_2_ hollow microspheres and Fe-doped CeO_2_ nanoparticles.

Thus, the advantages of HSMOs with respect to the photocatalytic degradation of pharmaceuticals and organic pollutions are given as follows: (1) the addition of substance promoted the separation of electron and hole pairs; (2) the large specific surface area provided more active sites; and (3) the abundant pore structure increased the probability of contact between the catalyst and pollution [217].

For AOPs’ treatment of methylene blue, Zhang et al. [212] prepared Fe_3_O_4_@MnO_2_ ball-in-ball hollow spheres (BBHs) with magnetic properties as the catalyst shown in Figure 18i–l. The improved catalytic activity of Fe_3_O_4_@MnO_2_ BBHs was ascribed to the synergistic effect of outer MnO_2_ nanosheets and the inner Fe_3_O_4_ hollow ball. And, the recyclability of the as-prepared catalyst was attributed to the magnetic property.

AOPs have been considered an effective means of water purification. The Fenton reaction is a specific kind of AOP. In addition, the Mn-doped Fe_3_O_4_ hollow microsphere [218], hollow spheres of Cu–CuFe_2_O_4_/SiO_2_ composite [219], bifunctional hollow mesoporous Fe^0^@C@MnFe_2_O_4_ [220], and the hollow sphere CuFe_2_O_4_ [221] have been prepared as Fenton-like catalysts for treating other dyes. These catalysts exhibited a superior catalytic activity for the removal of pollutants, good catalytic stability, and easy magnetic recovery due to the inherent characteristics of metal oxides and the advantages of a hollow structure, which could provide important instructions to rationally design and synthesize HSMOs for water pollution treatment.

## 3. Conclusions and Perspectives

HSMOs have piqued the interest of researchers as a novel type of structure. This structure takes advantage of the intrinsic properties of various metal oxides and fully exploits the various properties of the hollow structure, as well as the benefits of interfacial interaction and the synergistic effects between the metal oxides. Because metal oxide hollow structures have basic properties such as their high loading capacity, superior pore permeability, high specific surface area, abundant inner void space, and low density, they have a wide range of applications in environmental catalysis. We summarized their geometric morphology, metal oxide components, and interior/ulterior architecture from various levels and perspectives in different environmental catalysis processes in order to investigate the structure–performance correlation of HSMOs.

For environmental catalysis, the premise is to realize a highly catalytic performance by enhancing the probability of contact between the catalysts and the reactant, thus exposing more active sites. Countless HSMOs with properties such as a high specific surface areas, big pore volumes, and adjustable morphology have been developed. As the structure–activity relationship becomes clearer in this important field, the excellent catalytic performance is ascribed to the improvement in the properties in terms of magnetic properties, synergistic interaction, abundant defects, sufficient utilization of interior space, sufficient acid/base sites, good redox characteristics, abundant active oxygen, high storage/release capacity of oxygen, a large amount of active surface oxygen (O_S_), etc.

However, some aspects of HSMOs still need attention and deserve further study in order to optimize hollow structures in different applications, as described below:

(1) Specific laws of the synergistic effect between metal components of HSMOs. It is generally accepted that the synergistic effect is complex. However, the specific laws of the synergistic effect should be briefly summarized and then these novel laws should be generalized for practically useful synthetic approaches. For example, this could include the integration of different types of catalytic sites, such as acid, base, redox, oxygen vacancy, and other sites, using the variation of catalyst metal oxide compositions.

(2) Synthesis strategies need to be developed in order to reduce the process complexity and production cost. The synthesis of catalysts with hollow structures typically involves complex steps and elaborate formulations, which are more expensive than the conventional catalysts used in the industry. Therefore, the functional benefits offered by hollow-structured catalysts also come at a cost. In reality, in environmental catalysis, there are more substances that would cause catalyst poisoning and deactivation. To assess the real potential of catalysts, detailed techno-economic analyses and extensive benchmarking studies of the candidate and conventional catalysts are required.

(3) The rational design and optimization of the HSMOs’ shell porosity, thickness, and shell number are necessary. The catalytic kinetic behavior and molecular behavior control of HSMOs were regulated by these characteristics. The proper selection of microporous shells with a precise pore structure and size, increasing the number of shell layers, changing the thickness or curvature, etc., could promote selective reactions. The physical and chemical properties of HSMOs are investigated and tuned to enhance their interactions with the reacting gas molecules, thus effectively increasing the reaction rate.

Additionally, pioneering characterization technologies are highly preferred and urgently desired so that the aspects of HSMOs outlined above can be made much clearer with the use of advanced characterization data. Focusing on the structural characteristics of HSMOs, expounding the constitutive relationship between the component composition and its performance could have a guiding significance for the further optimization of the structure of the material, and it is expected to be widely used in the preparation of other HSMO materials. In the future, we can predict that the development of HSMOs for environmental catalysis will eventually intersect with various state-of-the-art fields.

## Figures and Tables

**Table 1:** Summary of the application of HSMOs in the elimination of environmental pollutants.

Items	Details
Automobile and stationary sources emission	Catalytic oxidation of CO
	NH_3_-SCR removal of NO_x_
	Catalyst for automobile three-way catalytic (TWC) reaction
	Catalyst for diesel oxidation catalytic (DOC) reaction
Volatile organic compounds (VOCs)	Catalytic oxidation of toluene
	Catalytic oxidation of vinyl chloride (VC)
	Catalytic oxidation of formaldehyde (HCHO)
Greenhouse gases	Catalytic conversion of CO_2_
	Catalytic conversion of CH_4_
Other potential pollutants	Hydrogenation of 4-nitrophenol (4-NP)
	Catalytic oxidation of 1,2-dichlorobenzene (o-DCB)
	Catalytic oxidation of dyes (e.g., acid orange 7(AO7), methylene blue)
	Photocatalytic degradation of pharmaceuticals (e.g., aceta-minophen, norfloxacin (NOR), tetracycline (TC), and ciprofloxacin)
	Photocatalytic degradation of organic pollutions (e.g., phenol)

**Table 2:** Pure CeO_2_ with various hollow structures and the catalytic performances of their CO catalytic oxidation.

Synthesis Method	S_BET_ (m^2^ g^−1^)	Catalytic Performance	Morphology	Ref.
One-pot template-free route	14.7	T_50_ = 280 °C	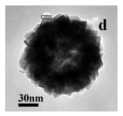	[46]
Hydrothermal process	22.0	—	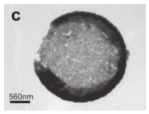	[47]
Template-free method	—	T_50_ < 270 °C	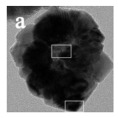	[49]
Surfactant-assisted solvothermal synthesis	74.0	—	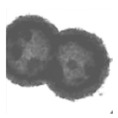	[54]
Self-template hydrothermal synthesis	36.7	—	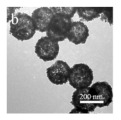	[67]
Template-free method	19.6	—	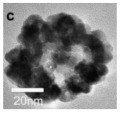	[69]
Template-free method	106.4	T_80_ < 310 °C	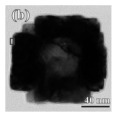	[63]
Solvothermal or hydrothermal route	147.6	T_95_ = 250 °C	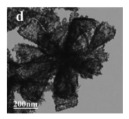	[64]
Ultrasonic-spray-assisted synthesis	75.8	T_100_ = 280 °C	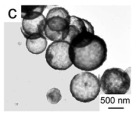	[66]
Yeast cells as templates	38.7	T_90_ = 372 °C	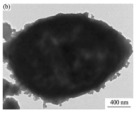	[65]
One-step liquid phase reaction	128.0	T_100_ = 170 °C	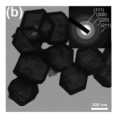	[68]

**Table 3:** The binary or multiple CeO_2_-based materials with various compositions, hollow structures, and catalytic parameters for CO catalytic oxidation.

Doped Metals	Material	Synthesis Method	S_BET_ (m^2^ g^−1^)	Total CO Conversion Temperature	Morphology	Ref.
Co	Co_3_O_4_-CeO_2−x_	Sequential templating approach	55.2	166.9 °C	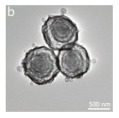	[70]
	Co_3_O_4_-CeO_2_	Self-templating method	44.8	145 °C	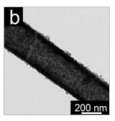	[71]
Cu	(Cu doping) CeO_2_	One-step solvothermal process	165.5	21 °C	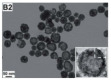	[74]
	CeO_2_-CuO_x_	Self-assembled approach	98.7	112 °C	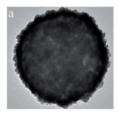	[75]
	CuCe-L	Aerosol-assisted synthesis	48.0~58.6	120 °C	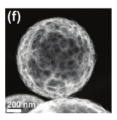	[76]
	CuO@CeO_2_	Surface Etching Strategy	36.0	—	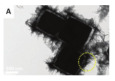	[77]
	CuO@CeO_2_	Template-free synthesis	90.0	60 °C	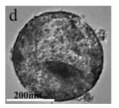	[78]
	CuO/CeO_2_-8%	Two-step route	24.9	130 °C	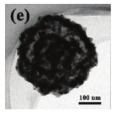	[79]
	Ce-MOF CeO_2_-CuO	Assistance of selective etching	86.7	98 °C	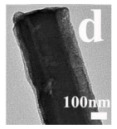	[80]
	CeO_2_-MO_x_ (M = Cu, Co, Ni)	Wet-chemical approach	—	160 °C	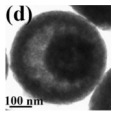	[81]
Mn	Ce–Mn Binary Oxide	Interfacial reaction-directed synthesis	202.0	T_50_ = 120 °C	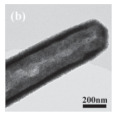	[55]
	MnO_2/_CeO_2_-MnO_2_	Sacrificial templates	103.1	206 °C	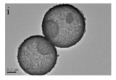	[82]
	CeO_2_@MnO_2_	Wet-chemical synthetic strategy	98.3	230 °C	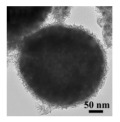	[83]
	CeO_2_-MnO_x_	Hard template-assisted solution combustion	115.2	160 °C	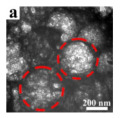	[84]
	Mn_2_O_3_@CeO_2_	Wet-chemical process	54.5	220 °C	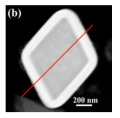	[85]
	CeO_2_-MnO_x_	Pyrolyzing Ce–Mn coordination polymers	77.8	~250 °C	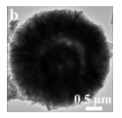	[86]
Fe	Fe_2_O_3_/CeO_2_	PB-based wet chemical approach	73.9	~230 °C	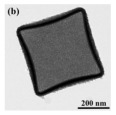	[87]

**Table 4:** CeO_2_-based hollow structure doped with noble metals with various compositions, structures, and their catalytic performances of CO catalytic oxidation.

Doped Noble Metal	Material	Synthesis Method	S_BET_ (m^2^ g^−1^)	Catalytic Performance	Morphology	Ref.
Pd	Pd@CeO_2_	Template-assisted and solvothermal alcoholysis strategy	73.3	T_90_ = 2 °C	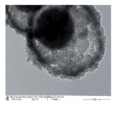	[92]
	h-Pd-CeO_2_ NCSs	Polymer-templated synthesis	59.3	T_80_ = 130 °C	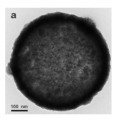	[91]
	MnO_2_-Pd-CeO_2_	Multi-assembly method	128.0	T_90_ = 90 °C	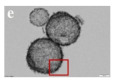	[93]
Au	Au/CeO_2_-ZnO	Chemical reaction	32.4	T_100_ = 60 °C	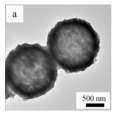	[94]
	Au/CeO_2_	One-step template-free strategy	145.0	T_92_ = 25 °C	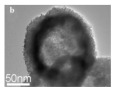	[95]
	Au/CeO_2_	Template-free method	23.9	T_90_ = 185 °C	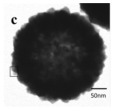	[96]
	Au@CeO_2_	In situ redox reaction	—	T_100_ = 21 °C	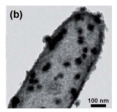	[97]
	Au@CeO_2_-ZrO_2_	Electrostatic attraction-induced deposition method	—	T_100_ = 130 °C	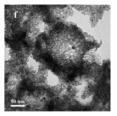	[98]
	Au/CeO_2_	Hard template synthesis method	77.8	T_100_ = 81 °C	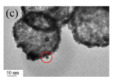	[3]
	Au/CeO_2_	Conventional solvothermal+ method auto-redox method	—	T_100_ = 73 °C	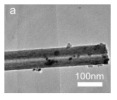	[99]
Pt	Pt_encap_/CeO_2_	Template-based procedure	—	—	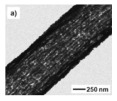	[57]
	CeO_2_-Pt	Interfacial reactions	62.3	T_100_ = 93 °C	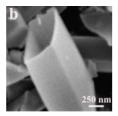	[100]
	Pt/CeO_2_	One-pot template-free solvothermal method	190.1	T_100_ = 155 °C	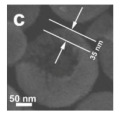	[101]
	Pt/CeO_2_@SiO_2_	Microemulsion method	146.2	T_100_ = 162 °C	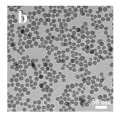	[102]

**Table 5:** Other HSMOs with various compositional, structure, and their catalytic performances of CO catalytic oxidation.

Materials	Synthesis Method	S_BET_ (m^2^ g^−1^)	T_100_ of 100% CO Conversion	Morphology	Ref.
α-Fe_2_O_3_ hollow microspheres	Ultrasonic-spray-assisted synthesis method	49.3	320 °C	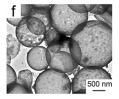	[66]
Co_3_O_4_ hollow microspheres	Ultrasonic-spray-assisted synthesis method	37.6	260 °C	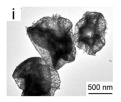	[66]
H-Co_3_O_4_@H-C	Reduction–oxidation pyrolysis process	104.0	130 °C	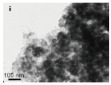	[105]
Hollow nanostructure Co_3_O_4_	Self-sacrificial template strategy	40.6	130 °C	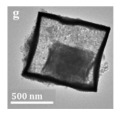	[106]
Core–shell nanostructure Co_3_O_4_	self-sacrificial template strategy	56.1	90 °C	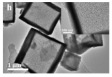	[106]
Au/α-Fe_2_O_3_-Hollow Catalysts	Hydrothermal–thermal decomposition process	10.9	—	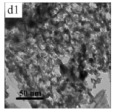	[107]
Hollow In_2_O_3_@Pd–Co_3_O_4_ core/shell nanofibers	Coaxial electrospinning	30.0	57 °C	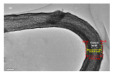	[108]
MnO_2_–Co_3_O_4_ hollow spheres	“Kirkendall effect” method	123.0	135 °C	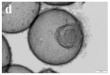	[109]

**Table 6:** Summary of toluene oxidation over the reported metal oxide catalysts with hollow structures.

Catalysts	Synthesis Method	S_BET_ (m^2^ g^−1^)	Reaction Conditions	Catalytic Performance			Morphology	Ref.
			Toluene Concentration, Weight Hourly Space Velocity (WHSV)	T_90_ (°C)	T_100_ (°C)	Ea (kJ mol^−1^)		
CeO_2_ hollow sphere	Hydrothermal methods	130.2	1000 ppm, 48,000 mL g^−1^ h^−1^	207	—	55.0	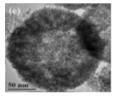	[147]
Hollow Co_3_O_4_ polyhedral nanocages	Thermal treatment of ZIF-67 templates	74.3	12,000 ppm, 21,000 mL g^−1^ h^−1^	259	280	77.9	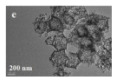	[152]
Flower-like MnO_2_ hollow microspheres	Interface reaction method	214.0	3000 ppm, 15,000 mL g^−1^ h^−1^	237	—	—	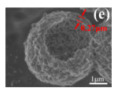	[151]
Manganese oxide polyhedra with hollow morphologies	Hydrothermal route	90.0	1000 ppm, 32,000 mL g^−1^ h^−1^	—	240	—	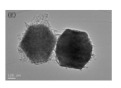	[153]
Pt/ZrO_2_ (0.57)	Modified Stöber process	285.0	1000 ppm in a total air flow of 100 mL min^−1^	172	—	—	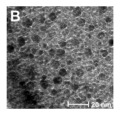	[144]
Pt/H-MnO_2_	Carbon spheres template method	54.0	1000 ppm, 60,000 mL g^−1^ h^−1^	180	—	—	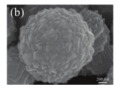	[154]
Nanocage-shaped Co_3−x_Zr_x_O_4_ loaded with Pt	Template method	23.5	50 ppm, 36,000 mL g^−1^ h^−1^	165	—	66.2	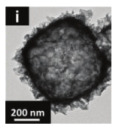	[20]
Pd-Modified NiCoO_x_ hollow nanospheres	Hard template method	162.1	500 ppm, 36,000 mL g^−1^ h^−1^	—	190	—	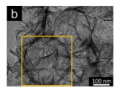	[6]
hollow microsphere CuMnO_x_	One-pot preparation	193.3	1000 ppm, 30,000 mL g^−1^ h^−1^	237	—	55.7	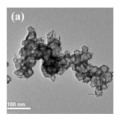	[155]
MnCeO_x_–OH hollow structure	Carbon spheres as hard templates	88.4	1000 ppm, 36,000 mL g^−1^ h^−1^	237	—	98.9	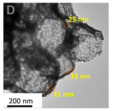	[156]
Ce_0.03_MnO_x_ hollow microsphere	Redox co-precipitation method	51.2	1000 ppm, 20,000 mL g^−1^ h^−1^	—	225	90.4	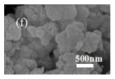	[157]
Hollow Mn_x_Co_3−x_O_4_ Polyhedron	Controlling heating rates	59.7	3000 ppm, 30,000 mL g^−1^ h^−1^	188	195	57.4	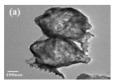	[158]
Hollow CoInO_x_ nanocube	SiO_2_ template strategy	36.0	3000 ppm, 30,000 mL g^−1^ h^−1^	178	—	41.6	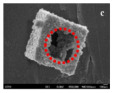	[150]

**Table 7:** Summary of catalytic activities in the VOC oxidation of reported metal oxide catalysts with hollow structures.

Catalysts	Synthesis Method	Textural Properties		Reaction Conditions	X (%)	T (°C)	Ref.
		S_BET_ (m^2^g^−1^)	Pore Volume (cm^3^g^−1^)				
K_x_MnO_2_ hollow nanospheres	Soft chemistry route	40.7	0.09	100 ppm HCHO, 20 vol % O_2_, GHSV = 50,000 h^−1^	80	100	[167]
MnO_2_ hollow spheres	Hard templating method	104.0~236.0	0.40~0.80	100 ppmv HCHO in dry air, GHSV 30,000 h^−1^	99.7	90	[23]
Au/MnO_2_ hierarchical hollow microsphere	Hydrothermal method and sol-gel method	52.3	0.16	200 ppm HCHO in air, GHSV 30,000 mL⋅g^−1^_cat_⋅h^−1^	59.2	25	[168]
Pt/C@MnO_2_ composite hierarchical hollow microspheres	Hydro- thermal method with hollow carbon spheres as a sacrificial template	153.0	0.37	HCHO solution (38% mass concentration)	90.5	__	[2]
Hierarchical Pt/WO_3_ nanoflakes assembled hollow microspheres	Solution method	23.0	0.12	HCHO solution (38% mass concentration), 260 ppm HCHO concentration	97	__	[165]
Hierarchically macro-mesoporous Pt/γ-Al_2_O_3_ composite hollow microspheres	Chemically induced self-transformation method	114.0	0.37	HCHO solution (38%)	__	__	[169]
Hierarchical Pt/NiO hollow microspheres	Template-free approach	50.8	0.11	HCHO solution (38%)	__	__	[170]
Pt/CoSn(OH)_6_ hollow nanoboxes	__	__	__	HCHO solution (38%), ~180 ppm HCHO concentration	80.1	__	[171]
Hollow chains mesoporous Pt/TiO_2_ (R_Pt-nominal_ were 0.5 wt%)	Microwave–hydrothermal route	132.0	0.29	HCHO solution (38%)	__	__	[145]
Fe_2_O_3_@SnO_2_ core–shell nanospindles	__	108.0	0.18	HCHO aqueous solution (38 wt%, contains 10–15 wt% methanol)	95.99	__	[172]
RuCoO_x_/Al_2_O_3_ hollow microspheres	Soft-template method	193.0	0.39	Gas containing 0.1% vinyl chloride in air, weight hourly space velocity (WHSV) of 30,000 mL·g^−1^·h^−1^	90	345	[166]

## Data Availability

Data are contained within the article.
